# Climatological and Epidemiological Conditions Are Important Factors Related to the Abundance of *bla*_KPC_ and Other Antibiotic Resistance Genes (ARGs) in Wastewater Treatment Plants and Their Effluents, in an Endemic Country

**DOI:** 10.3389/fcimb.2021.686472

**Published:** 2021-08-13

**Authors:** Erika A. Rodríguez, Nancy J. Pino, J. Natalia Jiménez

**Affiliations:** ^1^Línea de Epidemiología Molecular Bacteriana, Grupo de Investigación en Microbiología Básica y Aplicada (MICROBA), Escuela de Microbiología, Universidad de Antioquia, Medellín, Colombia; ^2^Grupo Diagnóstico y Control de la Contaminación (GDCON), Sede de Investigación Universitaria, Universidad de Antioquia, Medellín, Colombia

**Keywords:** antibiotic resistance genes, wastewater treatment plants, quantitative PCR, *bla*_KPC_, carbapenemases gene

## Abstract

Several physicochemical and season factors have been related to the abundance of antibiotic resistance genes (ARGs) in wastewater treatment plants (WWTPs), considered hotspots of bacterial resistance. However, few studies on the subject have been carried out in tropical countries endemic for resistance mechanisms such as *bla*_KPC._ In this study, the occurrence of ARGs, particularly *bla*_KPC_, was determined throughout a WWTP, and the factors related to their abundance were explored. In 2017, wastewater samples were taken from a WWTP in Colombia every 15 days for 6 months, and a total of 44 samples were analyzed by quantitative real-time PCR. *sul*1, *sul*2, *bla*_KPC_, and *erm*B were found to be the most prevalent ARGs. A low average reduction of the absolute abundance ARGs in effluent with respect to influent was observed, as well as a greater absolute abundance of ARGs in the WWTP effluent in the rainy season. Factors such as temperature, pH, oxygen, total organic carbon (TOC), chemical oxygen demand (COD), and precipitation were significantly correlated with the absolute abundance of several of the ARGs evaluated. A generalized linear mixed-effects model analysis showed that dissolved oxygen and precipitation in the sampling day were important factors related to the absolute concentration of *bla*_KPC_ over time. In conclusion, the abundance of ARGs in the WWTP could be influenced by endemic conditions and physicochemical and climatological parameters. Therefore, it is necessary to continuously monitor clinical relevant genes in WWTPs from different global regions, even more so in low-income countries where sewage treatment is limited.

## Introduction

Antimicrobial resistance constitutes an important and multifactorial public health problem worldwide. Therefore, its study and control cannot focus only on hospital institutions since humans, animals, and various interconnected environmental habitats can contribute to the emergence, evolution, and spread of antimicrobial resistance (Hernando-Amado et al., 2019).

Accordingly, other environments such as wastewater treatment plants (WWTPs) have been considered for study and intervention since they are one of the main receptors for antibiotics, resistant bacteria, and antibiotic resistance genes (ARGs) from anthropogenic activities (Berendonk et al., 2015). Additionally, their effluents are one of the sources of these emerging pollutants, constituting a potential risk to human health (Hong et al., 2013; Berendonk et al., 2015; Berglund et al., 2015). In recent years, WWTPs have been evidenced as becoming a reflection of the problem of resistance in the community (Sims and Kasprzyk-Hordern, 2020).

It has been described that the type of WWTP process can have important effects on the bacterial populations present in the water. For example, WWTPs with activated sludge technology would accelerate the genetic exchange processes of ARGs among bacterial populations due to the density and diversity of the bacterial populations present. Moreover, these populations can be selected due to the continuous contact with antibiotics and become reservoirs of ARGs, increasing the possibility of the appearance and dissemination of new resistance determinants (Hong et al., 2013; Novo et al., 2013; Yang et al., 2013).

Likewise, several studies have presented specific characteristics of the WWTP related to physicochemical, environmental, and even climatological variables, which seem to influence the abundance of ARGs in the treatment plants and their effluents (Hong et al., 2013; Novo et al., 2013). However, despite the advances in this regard, the great variability of factors such as operational conditions, and the particularities of each WWTP, makes it necessary to search, monitor, and evaluate the abundance of ARGs in treatment plants and effluents in different regions at a global level.

In this sense, molecular tools such as quantitative real-time PCR (qPCR) represent great advantages by giving an approximation of the prevalence, abundance, and dissemination of ARGs (Allen, 2014; Berendonk et al., 2015) that allows an approximation of bacterial resistance behavior in a given population to be made. Additionally, the analysis of the distribution of ARGs throughout the plant, together with the evaluation of the physicochemical and environmental characteristics, allows information on factors that can influence the presence of ARGs in a particular WWTP.

In South America and particularly in Colombia, the bacterial resistance problem is alarming, and antibiotics are used indiscriminately by the populations. Colombia is considered endemic for antibiotics resistance mechanisms of clinical relevance such as *bla*_KPC_ carbapenemase production (Munoz-Price et al., 2013). In 2005, *bla*_KPC_ was detected for the first time in hospital isolates (Virginia Villegas et al., 2006); since then, *bla*_KPC_ prevalence has been increased in the hospitals, which has limited the available therapeutic options (Mojica et al., 2012; Munoz-Price et al., 2013). Recently, this resistance mechanism has been reported in Gram-negative bacilli from the WWTP evaluated in this study, evidencing its presence in wastewater (Rodríguez et al., 2020).

Although in South America, Brazil, Chile, Argentina, and Colombia have advanced in describing the situation of resistance bacteria in aquatic environments (Chagas et al., 2011; Picão et al., 2013; de Oliveira et al., 2017; Aristizábal-Hoyos et al., 2019; Leon-Felix et al., 2020; Rodríguez et al., 2020), only a few studies have been carried out that quantify the occurrence and abundance of ARGs (Santamaría, 2011; Bueno et al., 2019; Arsand et al., 2020; Bueno et al., 2020). There are no long-term studies that evaluated the effect or influence of the climatological conditions of tropical countries and physicochemical factors in the presence and abundance of ARGs in the WWTPs and effluents, including those ARGs of clinical importance as *bla*_KPC_.

Taking the aforementioned into account, the objectives of this work are I) to determine the presence and abundance of ARGs for β-lactams, tetracyclines, sulfonamides, macrolides, and quinolones in an activated sludge treatment plant and II) to establish the stages of the treatment plant process and environmental and climatological factors that could be related to the presence of resistance genes and, in particular, the *bla*_KPC_ gene in the WWTP over time because *bla*_KPC_ is one of the most relevant resistance mechanisms in hospital isolates of the city and is very important in the world.

## Materials and Methods

### Place and Sample Collection

A cross-sectional study was conducted in a WWTP in Antioquia. Wastewater entering the treatment plant is a mixture of domestic and industrial wastewater. The average flow rate of wastewater treated by the WWTP over 1 year is 1.8 m^3^/s (ca.150,000 m^3^/day) with a removal efficiency of 80% biological oxygen demand and 85% suspended solids. The WWTP uses aerobic-activated sludge process technology and effectively treats 80% of the river’s flow that enters the plant. Due to its location, the WWTP collects wastewater from several schools, universities, hospitals, business offices, shops and clubs, and some industries. It processes the wastewater of 614,410 inhabitants.

**Figure 1:**
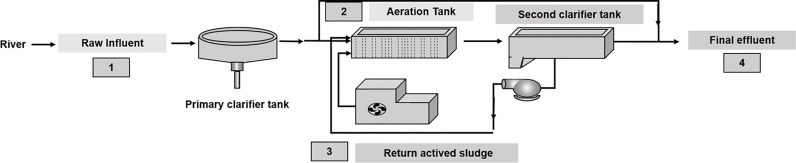
The figure shows the four sampling points of the wastewater treatment plant: raw influent, aeration tank, return activated sludge, and final effluent.

Samples were collected every 15 days over 6 months (January to July 2017) for 11 samplings at 4 sites, totalizing 44 samples. This period included dry and rainy seasons. The four specific sampling sites throughout the WWTP are raw influent (RI), aeration tank (AeT), return activated sludge (RS), and final effluent (FE) (**Figure 1**). To avoid effects associated with organic loading fluctuations, samples were collected every 15 days on the same day between 14:00 and 16:00. For each sample, 500 ml was collected and conserved at 4°C until laboratory analysis.

### Determination of Physicochemical Parameters and Atmospheric Conditions

The following physicochemical measurements were determined *in situ* and in triplicates with the use of Multiprobe (HACH HQ40d multi) (APHA et al., 2017): temperature (°C), pH, conductivity (μS/cm), dissolved oxygen (mg/L), and oxygen saturation (%). Other physicochemical parameters such as total solids (mg/L), chemical oxygen demand (COD), (mg/L), and total organic carbon (TOC) (mg/L) were measured according to the Standard Methods for Examination of Water and Wastewater (APHA et al., 2017). Additionally, atmospheric conditions such as seasons (dry and rainy) and water precipitation were obtained from the database of the Colombia Institute of Hydrology, Meteorology, and Environmental Studies (IDEAM - Instituto de Hidrología, 2017).

### DNA Extraction

A volume of 200 ml of each water sample was centrifuged at 13,000 rpm (17.950 g) for 15 min before DNA extraction. The supernatants were discarded, and the sediments were used in the extraction (approximately 0.1 to 0.33 g depending on the sample). For DNA extraction, the PowerSoil DNA Kit was used (Mo Bio, Carlsbad, CA, USA) (Xiong et al., 2014). The DNA concentration and purity were determined using a NanoDrop 2000 spectrophotometer (Thermo Scientific, Wilmington, DE, USA), and all DNA samples were stored at −20°C until further analysis.

### Quantification of ARGs

Quantitative PCR (qPCR) was used to quantify ARGs of clinical importance and that have been detected in aquatic environments, encoding resistance for beta-lactams (*bla*_KPC_, *bla*_NDM_, *bla*_OXA-48_, *bla*_CTX-M_, and *bla*_SHV_), macrolides (*erm*B), sulfonamides (*sul*1 and *sul*2), tetracyclines (*tet*W), and quinolones (*qnr*A). Also, the *16S rRNA* gene was analyzed to quantify total bacterial populations and normalize the abundance of ARGs in the collected samples.

Standard curves were prepared according to Marti et al. (2013). Each target gene was cloned into a pGEMR-T Easy Vector (Promega Corporation, Madison, WI) and transformed into *Escherichia coli* competent cells following the manufacturer’s protocol (Promega Corporation, Madison, WI). The amplicon-carrying pGEMR-T Easy Vector was purified with QIAprep Miniprep (QIAGEN, Germany) and linearized with *Bcu*I (*Spe*I) restriction enzyme (Thermo Scientific, Wilmington, DE, USA) before use. The plasmids concentration was determined using a NanoDrop spectrophotometer (Thermo Scientific, Wilmington, DE, USA), and the copy number was calculated according to Lee et al. (2006). The standard curve of each gene was generated by 10-fold dilution of plasmids carrying the target gene, ranging from 1 × 10^7^ to 1 × 10^1^ copies with three replicates. The amplification efficiencies of the standards for *16S rRNA* and ARGs ranged between 87.5% and 104.9%. The R2 values of the standard curves for *16S rRNA* and ARGs were in the range of 0.994 to 1.

All qPCR assays were performed with the CFX96 Touch™ Deep Well Real-Time PCR Detection System (Bio-Rad, Hercules, CA, USA), and PCR reactions were carried out in a 10-µl volume that contained 1 µl of template DNA (1 ng/µl), 1.6 µl of each primer (400 nM), 0.8 µl of nuclease-free water, and 5.0 µl of SsoFast EvaGreen Supermix (Bio-Rad, Hercules, CA, USA). **Table S1** summarizes primer sequences. The quantification was performed in triplicate for each sample within the same run together with the standard curves and DNA-free negative controls. The PCR inhibitors were eliminated by diluting the DNA with nuclease-free water. The specificity of qPCR assays was evaluated by the analysis of a melting profile. The limit of quantification (LOQ) was defined as the lowest number of target copies that can be reliably quantified. LOQ was determined as the minimum concentration of the target for which two replicates give a positive result with the coefficient of variability for Ct being no more than 0.5 Ct.

The ARG quantification results were expressed as absolute abundance and relative abundance. The absolute abundance of each ARG was expressed as the number of copies per milligram of the sample, and the relative abundance of each ARG was calculated by normalizing the absolute copy number of each gene to 16S rRNA gene copy numbers (ARG copies/*16S rRNA* copies).

### Statistical Analysis

According to data distribution, the description of the variable was made, calculating mean and standard deviation or median and interquartile ranges. ANOVA or Kruskal–Wallis tests were used to detect the statistically significant differences in the variables between the sampling points (p < 0.05).

The average reduction of the absolute abundance of ARGs in the different treatment stages concerning what entered to RI was evaluated using calculations [(average log reduction = log_10_ (mean gen copies influent)/(mean gen copies in respective WWTP stage)] (Pallares-Vega et al., 2019).

The relationship between the absolute abundance of ARGs, physicochemical parameters, and atmospheric conditions by sampling points was estimated by Spearman or Pearson correlations, and the level of significance was p-value < 0.05. Before this statistical analysis, the absolute abundance of each ARG was log_10_-transformed to meet normality assumptions.

Due to different samplings being carried out at the same time and place, a mixed-effects generalized linear model was applied to analyze factors related to the absolute abundance of *bla*_KPC_ in the WWTP during the study time. The outcome variable was the absolute abundance of *bla*_KPC_. Initially, a bivariate analysis was performed, where the variables included were season (dry and rainy), water temperature (°C), pH, conductivity (μS/cm), dissolved oxygen (mg*L-1), TOC (mg*L-1), COD (mgO2/L), total solids (mg*L-1), precipitation 1 day before sampling (mm), and precipitation in the sampling day (mm).

For multivariate analysis, variables that had a p-value of 0.25 were included and, considering that Colombia is a tropical country, the seasonal variables (dry and rainy) and precipitation in the sampling day also were included. A mixed-effects model with a random intercept family (gamma) and link (log) was used. Three models were generated and adjusted by sampling time. The likelihood approach was used to compare the models (Hall et al., 2000) based on the goodness-of-fit index as per the Akaike information criterion and the Bayesian information criterion (van den Hout et al., 2013). The lowest Bayesian information criterion was accepted as appropriate. Significant differences were reported at a p<0.05 level. The statistical analyses were carried out using the Stata v.14.0 software package (StataCorp LP, College Station, TX).

## Results

### Physicochemical Parameters and Atmospheric Conditions

**Table 1** shows the physicochemical parameters and atmospheric conditions determined during the study. The values found for the physicochemical parameters evaluated in the 6 months of the study complied with the values established locally on the requirements of specific wastewater discharges in surface water bodies and public sewerage systems (Resolution 0631 of 2015) (Ministry of Environment and Sustainable Development Colombia, 2015).

**Table 1:** Characteristic of wastewater and atmospheric conditions.

Characteristic	Raw influent (n=11)	Aeration tank (n=11)	Return activated sludge (n=11)	Final effluent (n=11)
**Water temperature (°C)^b^**	25.2 (0.79) Min (24) Max (26)	25.25 (1.18) Min (23.7) Max (27)	NA	24.8 (1) Min (23.4) Max (26.8)
**pH^a^**	7.68 (7.47–9.36)	7.325 (7.23–7.6)	NA	7.58 (7.41–8.03)
**Conductivity (μS/cm)^b^**	999.9 (277.7)	98.8 (212)	NA	923.8 (117.825)
Dissolved Oxygen (mg*L^-1^)^b*^	2.50 (1.03)	0.45 (0.42)	NA	4.5 (0.5)
**Oxygen saturation (%)^a^** ^*^	37 (21–41)	4.8 (2.8–8.2)	NA	63.4 (61.4–70.4)
TOC (mg*L^-1^)^b^	97.67 (85.82)	NA	NA	44.3 (29.9)
COD (mg O_2_/L)^b *^	692.7 (110.73)	NA	NA	267.7 (79.56)
Total solids (mg*L^-1^)^b*^	892.15 (128.37)	NA	NA	546.5 (68.74)
Suspended solids (mg*L^-1^)^a*^	248 (205–386)	956 (832–1364)	NA	110 (42–166)
**Precipitation 1 day before sampling (mm)^a^**	1.2 (0–10.9)	1.2 (0–10.9)	1.2 (0–10.9)	1.2 (0–10.9)
**Precipitation in the sampling day (mm)^a^**	1.9 (0–6.8)	1.9 (0–6.8)	1.9 (0–6.8)	1.9 (0–6.8)

Characteristics of wastewater and sludge samples in 11 months. IQR, interquartile range; COD, chemical oxygen demand; TOC, total organic carbon. **^a^**Median (IQR), maximum (MAX), minimum (min), **^b^**mean (SD). *Significant differences (p ≤ 0.05) between the points. NA, not applicable.

The treatment plant was observed to be operating efficiently in the reduction of organic matter and the improvement of water quality. Regarding the atmospheric conditions, 5 of the 11 samplings were carried out during the dry season and 6 during the rainy season. This classification was based on the one carried out for 2017 by the Colombia Institute of Hydrology, Meteorology, and Environmental Studies (IDEAM - Instituto de Hidrología, 2017).

### The Occurrence of ARGs in the WWTP

The occurrence of ARGs (*su*l1, *su*l2, *erm*B, *bla*_KPC_, *bla*_NDM_, *bla*_OXA-48_, *bla*_CTX-M_, *bla*_SHV_, *tet*W, and *qnr*A) was determined in 44 residual water samples (11 samples per point) during a 6-month period.

**Figure 2:**
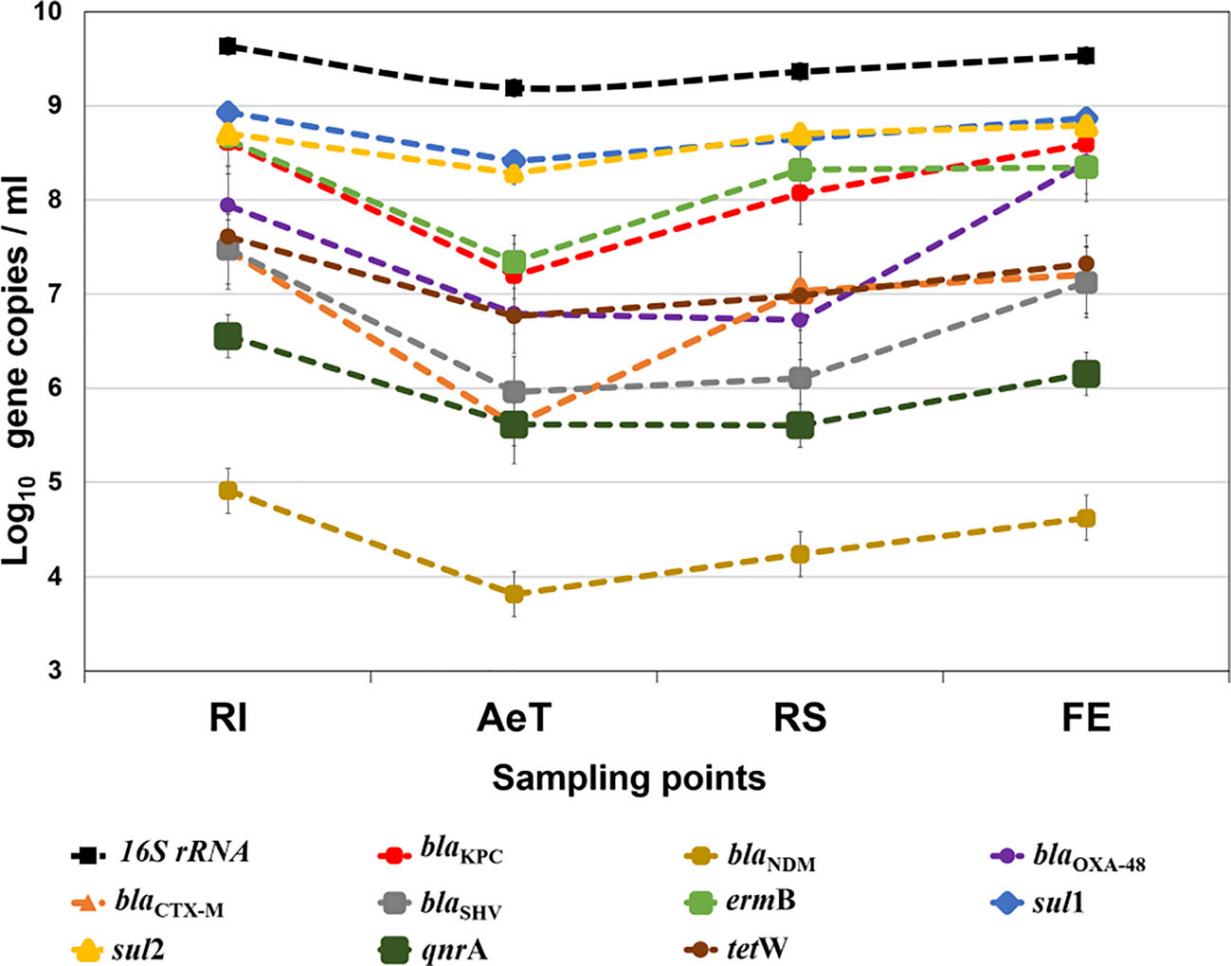
Absolute concentration of the *16S rRNA* gene and ARGs in the WWTP. The data represent the average concentrations of ARGs. The results are expressed in log_10_ copies per ml. Significant differences were found in the distribution of the ARGs between the sampling points except for the *sul*1, *sul*2, and *erm*B genes. RI, raw influent; AeT, aeration tank; RS, return activated sludge; FE, final effluent.

The results are summarized in **Table S2** and **Figure 2**. Regarding the number of copies of the bacterial *16S rRNA* gene, the average was 2.89 × 10^9^ copies/ml. Regarding the absolute abundance of ARGs, among the most prevalent were *sul*1, *sul*2, *bla*_KPC_, and *erm*B. The *sul*1 and *sul*2 genes were found in average concentrations of 5.74 × 10^8^ and 4.54 × 10^8^ copies/ml, respectively, the *bla*_KPC_ gene showed an average of 2.33 × 10^8^ copies/ml, and the *erm*B gene was found in an average concentration of 2.03 × 10^8^ copies/ml. Concerning other ARGs, it was detected in descending order of abundance: *bla*_OXA-48_ gene (8.74 × 10^7^ copies/ml), *tet*W gene (1.92 × 10^7^ copies/ml), *bla*_CTX-M_ gene (1.42 × 10^7^ copies/ml), *bla*_SHV_ gene (1.14 × 10^7^ copies/ml), *qnr*A gene (1.22 × 10^6^ copies/ml), and *bla*_NDM_ gene (2.99 × 10^4^ copies/ml).

The genes *sul*1, *sul*2, *bla*_KPC_, *tet*W, and *bla*_CTX-M_ were present in all the samples evaluated. Conversely, the genes *erm*B, *bla*_OXA-48_, and *bla*_SHV_ were detected in 36 of the 44 samples analyzed (**Table S3**), whereas the *bla*_NDM_ and *qnr*A genes were the least detected found in 15 and 31 of the 44 samples evaluated, respectively.

**Figure 3:**
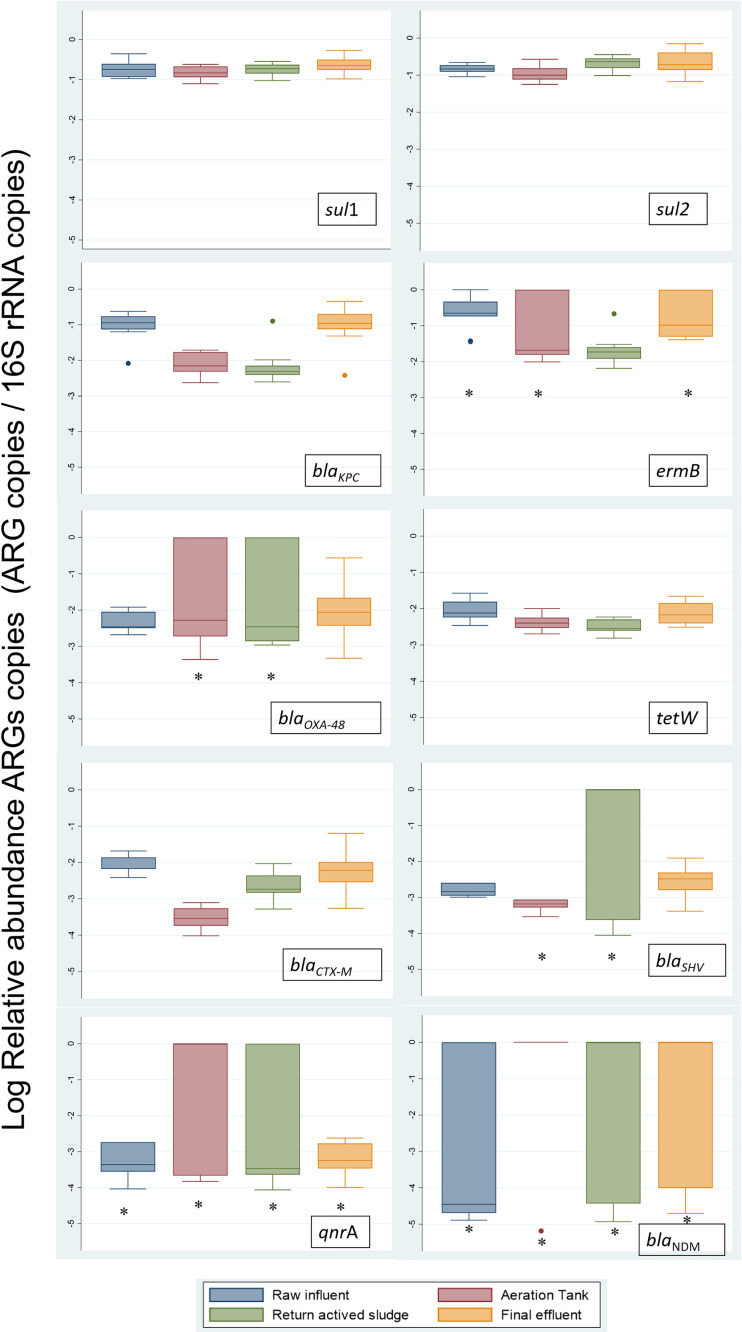
Relative abundance of ARGs in the WWTP. Within the box plot chart, the crosspieces of each box plot represent (from top to bottom) maximum, upper quartile, median (black bar), lower quartile, and minimum values. *erm*B, *bla*_KPC_, *bla*_NDM_, *bla*_OXA-48_, *bla*_CTX-M_, *bla*_SHV_, *tet*W, and *qnr*A showed a statistically significant difference between points (*P*<0.05). *ARGs with minimum values below the detection limit (*b.d.l*).

The relative abundance of ARGs normalized to the *16S rRNA* (ARG copies/*16S rRNA* copies) revealed trends in ARGs distribution (**Figure 3**). It was observed that the *sul*1, *sul*2, and *bla*_CTX-M_ genes’ relative concentration decreased in the AeT and increased in the RS and FE. In the *bla*_KPC_, *tet*W, and *erm*B genes, it was found that the relative abundance of these decreased in the secondary treatment and increased in the FE, sometimes even in higher concentration than in the RI. In the case of *bla*_OXA-48_, *bla*_SHV_, *qnr*A, and *bla*_NDM_, great variability was found in the relative abundance of these genes. The *bla*_OXA-48_ gene analysis showed a decrease through the plant but an increase in the FE. The *bla*_SHV_ gene increased in the RS and FE, and, in *qnr*A and *bla*_NDM_ genes, very similar values were observed at all the points of the WWTP.

When comparing the differences in the ARG distribution between the points of the plant evaluated, significant differences were detected in most ARGs except for *sul*1, *sul*2, and *erm*B (**Table S2**, **Figure 3**).

### The Reduction the Absolute Abundance of ARGs With Respect to Raw Influent

**Figure 4:**
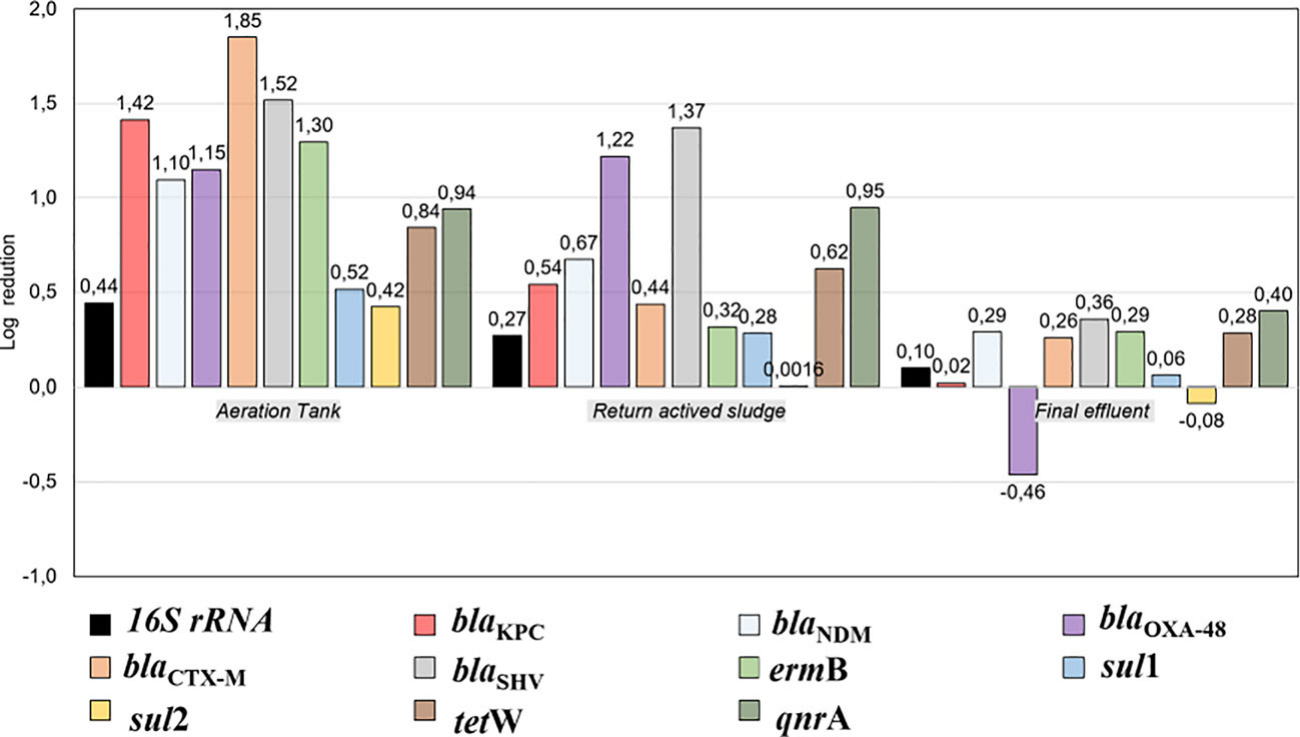
Reduction of concentration of *16S rRNA* and ARGs in the stages of the WWTP in comparison with raw influent [average log reduction = log_10_ (mean gen copies influent)/(mean gen copies in respective WWTP stage)]. RI, raw influent; AeT, aeration tank; RS, return activated sludge; FE, final effluent.

The average reduction of the absolute abundance of genes in the different treatment stages sampled concerning what entered to RI was evaluated (**Figure 4**, **Table S4**). A logarithmic reduction for the *16S rRNA* gene was observed mainly in the AeT (0.44 ± 0.62 logs), which means an average decrease of 63.9% of the gene; however, when the FE is analyzed, the average log reduction is only 20.3% (0.10 ± 0.12 logs).

Regarding the ARGs, it was observed that, in the AeT, the highest percentage of reduction was for the *bla*_CTX-M_ gene (98.59%, 1.85 ± 0.12 logs) and the lowest was for *sul*2 (62.23%, 0.42 ± 0.44 logs). In the RS, the highest reduction percentage was for *bla*_SHV_ (95.75%, 1.37 ± 1.27 logs) and the lowest was for *sul*2 with a percentage of 0.36%. Finally, in the FE, the average reduction, in general, was low, showing a greater percentage of reduction in the *qnr*A gene (60.21%, 0.40 ± 0.70 logs) and no reduction in the *bla*_OXA-48_ and *sul*2 genes, which, instead of decreasing, increased in the FE, mainly *bla*_OXA-48_ (**Figure 4**, **Table S4**).

### Relationship Between Physicochemical Parameters and Atmospheric Conditions and Absolute Abundance of ARGs Throughout the Treatment Plant

To establish the physical–chemical variables that could be related to the absolute abundance of ARGs in the WWTP, a correlation analysis (Pearson or Spearman) was used between physicochemical parameters and atmospheric conditions and the absolute abundance of ARGs at each point of the plant (**Table 2**). In general, correlations were found for most of the ARGs in at least one of the points of the plant except for the *bla*_NDM_ and *qnr*A genes. All correlation analyses are found in **Table S5 **and** Supplementary Figures 1–3**.

**Table 2:** Statistical estimation correlation between ARGs, physicochemical parameters, and atmospheric conditions.

Stage of the WWTP	Characteristic								
	Water temperature (°C)	pH	Dissolved Oxygen (mg*L^-1^)	Oxygen saturation (%)	TOC (mg*L^-1^)	COD (mgO_2_/L)	Suspended solids (mg*L^-1^)	Precipitation 1 day before sampling (mm)	Precipitation in the sampling day (mm)
***Raw influent***									
***bla_KPC_***	-0.259	-0.194	0.516	0.452	0.208	0.065	0.190	0.529	**0.651**
***bla*_OXA-48_**	0.527*	0.246	0.065*	0.058	**0.681**	0.718*	0.138	-0.106	0.054
***bla_SHV_***	0.090*	0.226	0.619*	0.538	0.540	0.109*	0.336	0.471	0.451
***sul*1**	-0.077	0.000	0.357	0,204	0.275	0.306	**0.717**	0.104	0.566
***sul2***	-0.521*	0.404	0.543*	0.404	0.609	0.069*	**0.640**	0.295	0.583
***tetW***	-0.266*	0.224	0.770*	**0.768**	0.270	-0.257*	0.312	0.535	0.549
***Aeration Tank***									
***bla_CTX-M_***	-0.13*	0.058	0.481*	0.318	NA	NA	0.426	0.680^*^	0.233
***ermB***	**0.607**	0.294	0.132	0.069	NA	NA	-0.202	-0.028	0.213
***sul2***	0.739*	0.373	NA	-0.075	NA	NA	0.522	-0.114	-0.150
***qnrA***	0.630	**0.810**	0.412	0.235	NA	NA	0.388	0.011	-0.301
***Final effluent***									
***bla_KPC_***	-0.451*	-0.118	0.119*	-0.103	0.112	0.103	-0.390	0.173	**0.683**
***bla_OXA-48_***	0.123*	0.109	-0.084*	-0.060	**0.672**	0.189	0.474	0.153	0.532
***bla_CTX-M_***	-0.587*	0.277	0.357*	0.235	0.312	0.128	-0.481	0.426	**0.699**
***bla_SHV_***	-0.426*	-0.122	0.357*	0.224	0.215	0.082	-0.233	**0.621**	**0.741**
***sul1***	-0.496*	-0.280	0.295*	0.127	-0.055	0.357	-0.143	0.339	**0.772**
***sul2***	-0.709*	-0.060	0.419*	0.179	0.114	0.179	-0.343	0.642^*^	**0.784**
***tetW***	-0.724*	-0.078	0.275*	0.039	-0.020	0.117	-0.383	**0.438**	**0.629**

Significant differences (p ≤ 0.05) are shown in dark gray and bold. The table shows only those variables in which correlations were detected.

Spearman or *Pearson correlations.

NA, not applicable.

The water temperature showed a positive correlation with the absolute abundance of the *erm*B (0.607) and *sul*2 (0.739) genes in the AeT. The TOC and COD parameters were positively correlated with the *bla*_OXA-48_ gene count in the RI (TOC (0.681)), COD (0.718)), and FE (TOC (0.672)).

Regarding dissolved oxygen, a positive correlation was found between the RI and the *bla*_SHV_ (0.619) and *tet*W (0.770) genes. *tet*W also showed a positive correlation with oxygen saturation (0.768) at this point in the plant.

The presence of suspended solids showed a positive correlation for the absolute concentration of the *sul*1 (0.717) and s*ul*2 (0.640) genes in the RI, and the pH presented a positive correlation with the *qnr*A (0.619) gene in the AeT.

Atmospheric conditions were also positively related to the absolute concentration of ARGs. Thus, precipitation 1 day before sampling was correlated with *bla*_CTX-M_ (0.680) in the AeT and *bla*_SHV_ (0.621), s*ul*2 (0.642), and *tet*W (0.438) in the FE. Additionally, the precipitation in the sampling day showed significant positive correlations with *bla*_KPC_ (0.651) in the RI and with *bla*_KPC_ (0.683), *bla*_CTX-M_ (0.699), *bla*_SHV_ (0, 741), *sul*1 (0.772), *sul*2 (0.7840), and *tet*W (0.629) in the FE.

**Figure 5:**
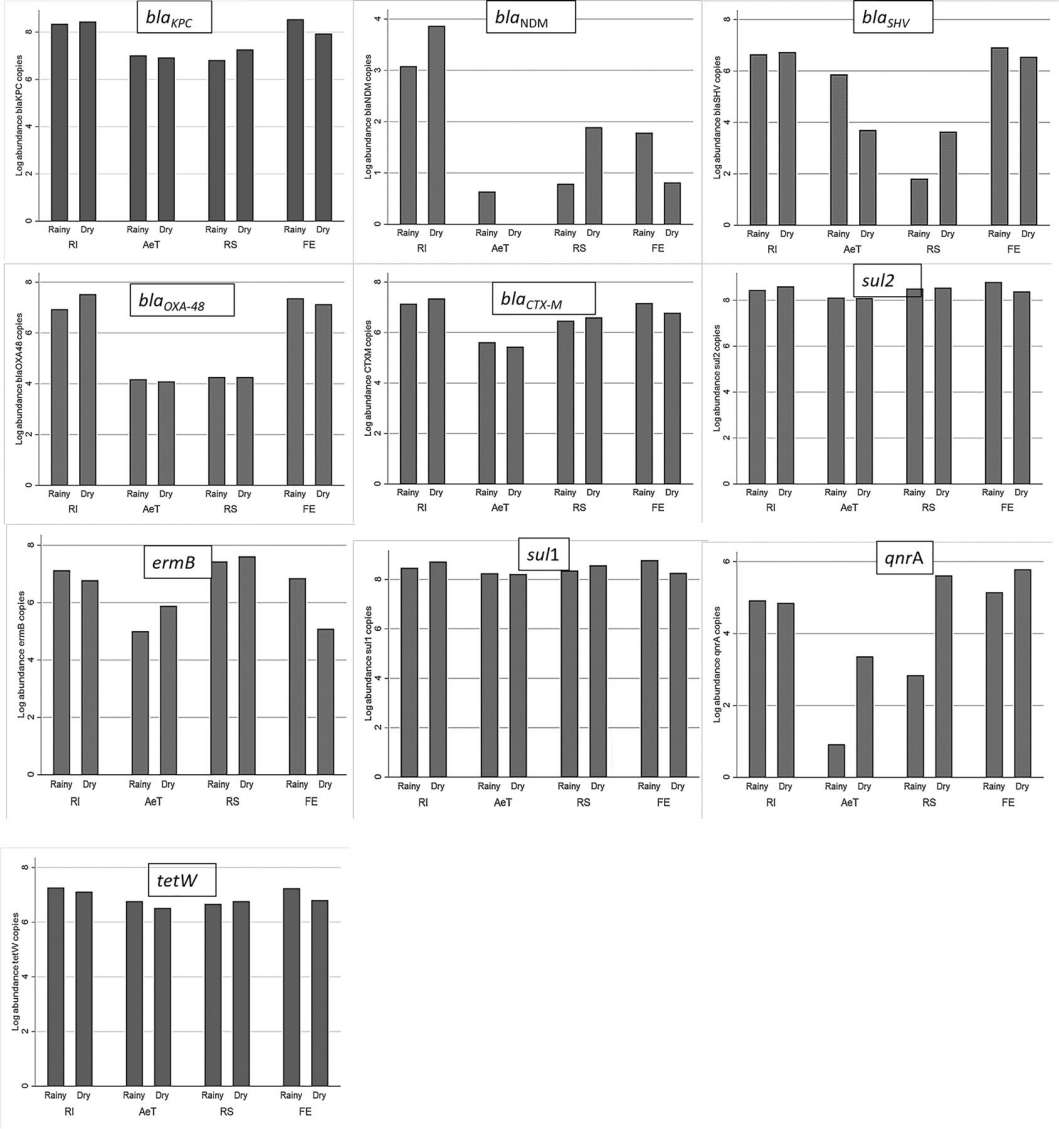
Absolute concentration of each ARG in samples collected from RI, AeT, RS, and FE in both rainy and dry seasons. The data represent the average concentrations of ARGs. The results are expressed in log_10_ copies per ml.

Considering the findings of correlations, it is evidenced by a direct relationship between precipitation and the absolute abundance of ARGs in the effluent of the plant. The effect of the seasons (dry and rainy) on the absolute concentration of ARGs at each point of the treatment plant was evaluated. It was found that the absolute concentration of the majority of the resistance genes showed a characteristic pattern: in the dry season, a greater absolute abundance of ARGs was observed in the RI and RS, whereas in the rainy season, a greater absolute abundance of ARGs was observed in the FE and AeT except for the *tet*W and *qnr*A genes (**Figure 5**).

### Factors Related to the Absolute Abundance of *bla*_KPC_ in the WWTP During the Study Time

**Figures S4** and **5** show the distribution of *bla*_KPC_ by season (**Figure 5**) and over the study period (**Supplementary Figure 4**) by sampling place. In both, a slight increase in the absolute abundance of *bla*_KPC_ is observed in the FE compared to the RI. A mixed-effects generalized linear model analysis was performed to analyze the factors related to the absolute abundance of *bla*_KPC_ during the study period. Two models were generated (**Tables S6, S7**, and **Table 3**). The model that showed the best fit is shown in **Table 3**. In the models, the variable related to the absolute abundance of *bla*_KPC_ during the study period was the increase in dissolved oxygen. When this variable was evaluated together with meteorological variables such as the season of the year (**Table S7**) or precipitation in the sampling day (**Table 3**), it was that for each increase in the concentration of dissolved oxygen (p <0.001) and precipitation in the sampling day (p <0.051), the absolute abundance of *bla*_KPC_ increased in the WWTP.

**Table 3:** Model 2. Factors related to the abundance of *bla*_KPC_ in the WWTP during the study time.

Factor	Crude				Adjusted			
	β	p-value	CI 95%		β	p-value	CI 95%	
Dissolved oxygen (mg/L)	0.746	0.000	0.390	1.103	0.675	**0.000**	0.328	1.022
Precipitation in the sampling day (mm)	0.018	0.630	-0.055	0.090	0.057	**0.051**	-0.002	0.116

Multilevel mixed-effects generalized linear model (GLM). Gama, β: model estimate. Adjusted by sampling time.

## Discussion

In this work, the presence and abundance of ARGs throughout a WWTP in a tropical country over 6 months was determined. This allowed a follow-up and evaluation of ARG concentration over time and an analysis of different factors related to the absolute abundance of ARGs in the plant. It is also one of the first studies in the region on the subject and focused on describing those factors related to the absolute abundance of *bla*_KPC_ in a WWTP, an endemic resistance gene in South America.

Our results show that the absolute abundance of ARGs in a WWTP is a dynamic and multifactorial process influenced by the specific operating conditions of each WWTP, the physicochemical parameters, the atmospheric conditions such as the rainy season, and possibly the epidemiology trends of the bacterial resistance in each region. These types of findings highlight the importance of continuous monitoring and epidemiological surveillance of the presence and abundance of ARGs in treatment plants and effluents in different regions globally, and more so in geographic areas such as South America where wastewater treatment is limited, and bacterial resistance represents a great threat to public and environmental health.

### The Presence and Abundance of ARGs in WWTPs

In the study, significant differences were found in the distribution of ARGs between the points of the plant. Due to the large number of reports on the *sul*1 and *sul*2 genes in the WWTP and their importance as markers of environmental contamination, the absolute abundance of these sulfonamide resistance genes was evaluated. The results showed a high abundance of *sul*1 and *sul*2 genes in the WWTP (*sul*1 5.74 × 10^8^ and *sul*2 4.54 × 10^8^ copies/ml) and corroborated previous findings in other WWTPs, where they describe them as the most abundant resistance genes (Pei et al., 2006; Wang et al., 2013; Calero-Cáceres et al., 2014; Mao et al., 2015; Cacace et al., 2019; Pallares-Vega et al., 2019; Paulus et al., 2020). The high absolute abundance of sulfonamide resistance genes in wastewater is due to the extensive use that has long been made of these antibiotics in human and animal practices (Aminov et al., 2001; Pei et al., 2006); besides, they are generally found in mobile genetic elements such as class 1 and class 2 integrons, which are frequently described in aquatic environments (Gillings, 2014; Koczura et al., 2016).

Likewise, in the treatment plant, the presence of *erm*B gene was screened. This macrolide resistance gene was detected at considerable absolute concentrations (2.03 × 10^8^ copies/ml). As with the *sul* genes, *erm*B gene is one of the most evaluated and found resistance genes in wastewater worldwide (Pallares-Vega et al., 2019; Paulus et al., 2020). Its presence in water has been associated with water pollution by antibiotics such as azithromycin and industrial effluents’ discharges (Milaković et al., 2019).

Another gene analyzed was *tet*W (resistance to tetracycline) that was detected at average concentrations of approximately 1.92 × 10^7^ copies/ml. In Colombia, a previous study conducted on water bodies from agricultural and livestock operations showed the presence of tetracycline resistance genes (Santamaría, 2011). This work shows the presence of tetracycline resistance genes in an urban WWTP in Colombia. The high abundance of these ARGs is explained by the wide use of tetracyclines, mainly in livestock activities. Likewise, *tet* genes are considered indicators of fecal contamination (Aminov et al., 2001; Pei et al., 2006), which explains their presence in the WWTP.

In the treatment plant, some genes were found to be in lower absolute abundance. One of these was the plasmid-mediated quinolone resistance (PMQR) determinant *qnr*A (1.22 × 10^6^ copies/ml). This gene confers resistance to quinolones, one of the most commonly prescribed antibiotics for human and animal infections. PMQR has been detected in bacterial isolates of hospital and municipal wastewater (Marti and Balcázar, 2013; Vaz-Moreira et al., 2016). The search for these determinants is important because these genes are associated with extended-spectrum beta-lactamases (ESBL) and plasmid-mediated cephalosporinases and favor the selection of additional chromosomally encoded quinolone resistance mechanisms (Poirel et al., 2008).

Likewise, clinically relevant ARGs *bla*_KPC_, *bla*_NDM_, *bla*_OXA-48_, *bla*_CTX-M_, and *bla*_SHV_ were evaluated in this study. The *bla*_CTX-M_ and *bla*_SHV_ genes were found in medium proportions in the WWTP (*bla*_CTX-M_ 1.42 × 10^7^ copies/ml and *bla*_SHV_ 1.14 × 10^7^ copies/ml), corroborating previous findings on beta-lactamase–producing *Enterobacteriaceae*, where these resistance genes were frequently detected.

Regarding genes that encode carbapenemase-type beta-lactamases (*bla*_KPC_, *bla*_NDM_, and *bla*_OXA-48_), the *bla*_KPC_ gene was detected in considerable absolute concentrations in the WWTP (an average of 2.33 × 10^8^ copies/ml). This finding coincides with previous results at the local level for our research group, where high endemicity of this resistance mechanism is reported in carbapenem-resistant Gram-negative bacilli isolated from hospitalized patients and the WWTP (Ocampo et al., 2016; Rodríguez et al., 2020). It also coincides with the global findings of countries such as China, where reports of high concentration *bla*_KPC_ in wastewater are increasingly frequent (Yang et al., 2016; Yang et al., 2017). However, the results are contrary to those reported in European countries, where a low abundance of *bla*_KPC_ is described (Cacace et al., 2019; Schages et al., 2020); among them is Germany, which reported other carbapenemases with higher frequency (Schages et al., 2020). These differences respond to the local epidemiology of bacterial resistance and justify the search and continuous surveillance of common ARGs in hospital settings. Likewise, the finding of the *bla*_KPC_ gene in the effluent indicates the risk of spread in the environment.

The other genes that encode carbapenemases, such as *bla*_NDM_ (2.99 × 10^4^ copies/ml) and *bla*_OXA-48_ (8.74 × 10^7^ copies/ml), had low occurrence and absolute abundance in the WWTP compared to *bla*_KPC_. The low absolute abundance found is again explained by the country’s epidemiology trends, where also a low frequency of these resistance genes has been observed in hospitals. Unlike what happens in Europe and Asia, these ARGs are frequently detected in hospitals and aquatic environments (Yang et al., 2016; Cacace et al., 2019; Schages et al., 2020). However, due to their clinical relevance, the *bla*_NDM_ and *bla*_OXA-48_ sporadic detection reveals the need to continue their follow-up and monitoring because, although in low circulation, they could become a critical bacterial resistance problem at the local level.

Generally, the analysis of the presence and absolute abundance of ARGs in the WWTP showed two relevant aspects. First, those ARGs frequently reported globally in WWTPs such as *sul*1, s*ul*2, and e*rm*B were also presented in abundance in the WWTP evaluated. This result agrees with the findings of Cacace et al. (2019) who describe few differences in biogeographic patterns with respect to the abundance of these genes in urban WWTP effluents. Therefore, they suggest that although *sul*1, s*ul*2, and e*rm*B are considered markers of the degree of antibiotics water pollution, their evaluation in urban WWTPs may not be helpful and their search could be more directed in rural environments. Second, ARG monitoring in WWTPs should be directed to clinical relevance ARGs with potential risk in public health, such as carbapenems-resistant genes. In addition to showing the impact of resistance in the population, clinical relevance ARG abundance, generally in wastewater, responds to bacterial resistance’s local epidemiology, which justifies their constant follow-up to detect the emergence of these ARGs in the population.

### ARGs: The Distribution and Reduction of ARGs Throughout the Different Points of WWTP

The distribution analysis of all ARGs throughout the plant, together with the reduction values of absolute abundance, gives an overview of the role of WWTPs in the elimination of ARGs (Pallares-Vega et al., 2019), relevant information to target wastewater treatment. Although the relative abundance of the ARGs evaluated throughout the plant was different, in all cases, an increasing trend of ARGs in the effluent was evident. These results were supported by the low percentage of absolute abundance reduction of bacterial *16S rRNA* and ARGs in the effluent, which were found at an even lower percentage than those reported in effluents of a conventional activated sludge WWTP (Czekalski et al., 2012; Rodriguez-Mozaz et al., 2015; Makowska et al., 2016; Rafraf et al., 2016; Pallares-Vega et al., 2019).

The finding of low bacterial *16S rRNA* removal alerts about the functioning of the WWTP in bacterial removal. This behavior has been reported in other studies, where the reduction of *16S rRNA* in the effluent has been described as insignificant, and an increase in this gene in the FE has been reported (Rafraf et al., 2016; Korzeniewska and Harnisz, 2018). However, this differs from other works, where removal percentages of bacterial *16S rRNA* of up to 98.2% in the effluent have been described (Pallares-Vega et al., 2019).

Regarding the distribution of the relative abundance of *bla*_KPC_, *bla*_OXA-48_, *tet*W, and *erm*B, it was observed that these genes decrease during treatment; good removal percentages were observed in tanks and sludge but increased in the effluent. These results may indicate several things: on one hand, the dispersion processes of ARGs from secondary treatment, where ARGs are released from bacteria that carry these ARGs by predation processes by bacterivorous protozoa or by the effect of treatment in which low hydraulic retention times or environmental phenomena such as rain (discussed later), or both, allow these genes to arrive from specific points of the plant to the FE. On the other hand, the specific functioning of the WWTP studied, where only 80% is treated, and the additional 20% is mixed with the FE, would explain the sudden increase in genes such as *bla*_KPC_, which showed a low relative abundance in the recirculating sludge but a sudden increase in the effluent. Although this WWTP design is uncommon, this result indicates the importance of treating 100% of the flow that enters the WWTP, which would help improve the removal of ARGs.

Different behavior was observed when the distribution of the relative abundance of *sul*1, *sul*2, and *bla*_CTX-M_ was analyzed: these genes decreased in tanks and increased in the sludge and effluent. These results agree with the removal analysis where low percentages were observed in sludge and effluent for genes such as *sul*2 (0.36%). Both results may show selective processes in recirculation activated sludge for these resistance genes by substances such as antibiotics, which are described as the main selective factors in treatment plants. These findings are consistent with the results of previous studies from the same treatment plant, where compounds such as sulfamethoxazole and trimethoprim were found in abundance in the WWTP effluent (Botero-Coy et al., 2018). Likewise, they may result from the lack of control in the use of antibiotics in the community in the country of study.

Finally, variability in the relative abundance of *bla*_OXA-48_, *bla*_SHV_, *qnr*A, and *bla*_NDM_ was observed throughout the study, which may be due to the sporadic and low detection of these genes in the WWTP. Therefore, it is difficult to generate hypotheses on the factors that would explain the distribution of these ARGs in the WWTP points evaluated.

### Environmental Factors Related to the Presence of Resistance Genes and in Particular of the *bla*_KPC_ Gene in WWTPs Over Time

Different physicochemical factors seem to influence the absolute abundance of ARGs in treatment plants and their effluents (Hong et al., 2013; Novo et al., 2013). Our results indicate how the particular operating conditions of WWTPs and weather conditions favor the presence of ARGs in the WWTP. It was found that variables such as water temperature, TOC, COD, oxygen, pH, and rainfall can probably affect the absolute abundance of some ARGs at specific points in the WWTP. All these factors directly affect bacterial growth, and some of the bacterial species that carry these resistance genes may be more sensitive to the changes produced by these variables, and, for this reason, they increase or decrease, which is directly reflected in the concentration of these genes in specific parts of the WWTP.

Previous studies have indicated an important effect of temperature on bacterial resistance (Manaia et al., 2018). A positive correlation between temperature and *erm*B and *sul*2 abundance was observed in the AeTs in this work. The correlation found may be due to an overgrowth of microorganisms typical of the microbial communities that carry these ARGs at this point in the plant. An opposite effect was observed in the influent and in the effluent, which is possibly explained because these points of the WWTP are bacterial transit points, where retention times are short and, therefore, the temperature does not have a significant effect on the growth of microorganisms.

Other parameters described to be related to the absolute abundance of ARGs in WWTPs are TOC and COD (Manaia et al., 2018), which, in this work, were correlated positively with the *bla*_OXA-48_ gene in the influent and the effluent. TOC and COD could be favoring the growth and survival of bacteria carrying *bla*_OXA-48_ in the influent and the effluent, considering that, in the study’s WWTP, a treatment is carried out on 80% of the flow and the other 20% of the water is untreated. A slight increase in the organic load (TOC and COD) in the FE is expected. This behavior could explain the low average reduction of *bla*_OXA-48_ in the effluent. Other studies have reported a relationship of TOC with other ARGs, including *erm*B and tetracycline resistance genes (Kim et al., 2007; Manaia et al., 2018).

With regard to the effect of weather conditions on the absolute abundance of ARGs in the WWTP, in general, few studies have explored these variables (Caucci et al., 2016; Koczura et al., 2016; Lamba and Ahammad, 2017; Pallares-Vega et al., 2019; Schages et al., 2020) and especially in tropical countries where the seasons are not distinctly defined. Interestingly, we found a positive relationship between atmospheric conditions such as precipitation 1 day before sampling and precipitation in the sampling day with the absolute abundance of *bla*_KPC_, *bla*_CTX-M,_ *bla*_SHV_, *sul*1, *sul*2, and *tet*W mainly in the effluent. This finding was confirmed with the analysis of the distribution of absolute concentration of ARGs according to the weather season (rainy or dry) and with a mixed-effects generalized linear model applied to analyze factors related to the absolute abundance of *bla*_KPC_. In these analyses, variables such as precipitation in the sampling day and the increase in dissolved oxygen could be related to the increased absolute concentration of *bla*_KPC_ in the WWTP during the study time.

Our results contradict what was described by Mao et al. (2015) and Lamba and Ahammad (2017) who did not find changes in the abundance of genes in WWTPs in rainy or dry seasons; however, they agree with what was previously described by other authors who have detected changes in abundance according to the time of the year or the presence of rain (Yang et al., 2013; Di Cesare et al., 2017; Sui et al., 2017; Schages et al., 2020).

For most of the ARGs, a greater absolute concentration was observed in the effluent in the rainy season. An increase in the abundance of ARGs in the effluent in the winter or rainy season in the WWTP and rivers has been previously reported (Yang et al., 2013; Caucci et al., 2016; Koczura et al., 2016; Di Cesare et al., 2017). The abundance of ARGs in rainy seasons has been related to the increase in the consumption of antibiotics in the populations (Caucci et al., 2016), which would lead to an increase in selective processes in the WWTP; however, in this case, this variable could not be determined.

Based on the analysis of the effect of the seasons on the absolute concentration of ARGs at each point of the WWTP, we hypothesize that the variations in the abundance of ARGs found in the different points of the plant during the seasons could be related not only to selective processes but also to dispersion processes and the dilution of ARGs. Thus, in the rainy season, the decrease in ARGs in the influent may be due to the dilution process of bacteria and ARGs that would reach the WWTP in lower concentrations due to the increase in the riverbed. Likewise, the increase in ARGs in the effluent may be due to an increase in the hydraulic load of the WWTP, which would lead to a process of less hydraulic retention time in tanks. Therefore, the rain would favor dispersion phenomena of resistant bacteria and ARGs in tanks to the effluent. This hypothesis can be supported by the previous findings of Pallares-Vega et al. (2019) where it is described how a higher hydraulic load can disturb the treatment processes and affect in some way the efficiency of ARG reduction during treatment. Likewise, Di Cesare et al. (2017) described the effect of moderate rains on the abundance of ARGs in a river, finding that they increase significantly in rainy seasons (total ARGs: 24 times) concomitantly with microbial aggregates that could detach possibly due to disturbances generated by rain (Di Cesare et al., 2017).

Because the generation and dissemination of antibiotic resistance in the environment is a complex process, and the result of the interaction of different variables that can be masked with correlation analysis, a mixed-effects generalized linear model analysis was performed to determine factors related to the absolute abundance of *bla*_KPC_ during the study time. Our results showed that the *bla*_KPC_ concentration increased concomitantly with the increase in the dissolved oxygen concentration and the precipitation in the sampling day. Rain is an important factor that can generate variations in the physicochemical parameters of the water, and it has been seen that, in high volumes of rain together with a rapid rate of flow, dissolved oxygen is significantly increased (Girardi et al., 2016). This would explain the relationship between these variables and their impact on the increase in *bla*_KPC_ in the WWTP. Similar analyses have shown that the abundance of ARGs has been related, in addition to rainfall, to the increase in total phosphorus, N-NH4, and microbial aggregates (Di Cesare et al., 2017).

Finally, in this study, all water samples were centrifuged for DNA extraction, and sediments were used. The methodology variation is due to the number of solids found in the samples analyzed and the particular WWTP operation, where a part of the FE is again mixed with the initial RI (20%). Therefore, there was an important amount of solids in some samples, which made the filtering method difficult. Although it is not a common method in wastewater studies, due to the conditions described, we consider it an adequate approximation of what is happening in the WWTP. All samples (including sludge) were processed under the same conditions and normalized, decreasing the noise that could be generated when the samples are processed with different extraction methods, whereby the results could allow a more precise comparison between the points of the plant. Likewise, due to changes in the methodology, the results were not directly compared with the ARG quantification of other studies. Nevertheless, more studies are needed to confirm the findings.

## Conclusions

The results of this work allowed the monitoring and evaluation of the treatment process in a WWTP and abundance of ARGs, including *bla*_KPC_, over a period of time. These suggested that the particular operating conditions of the WWTP, i.e., physicochemical factors (water temperature, TOC, COD, oxygen, and pH), the climatic conditions of tropical countries (rain and drought), and the prevalence of resistance mechanisms such as *bla*_KPC_ in local hospitals, could be related to the absolute abundance of ARGs in the WWTP. Further investigations should be conducted to explore other variables that could influence the abundance of ARGs in the WWTP and their effluents and could impact and be risk factors of antimicrobial resistance, such as the number of infected patients, self-medication, and consumption of antibiotics in the populations. Likewise, our results established the need to continue routine ARG monitoring in wastewater to detect new trends and threats.

## Data Availability Statement

The original contributions presented in the study are included in the article/**Supplementary Material**. Further inquiries can be directed to the corresponding authors.

## Author Contributions

ER and JJ contributed to the conception and design of the study. ER performed laboratory work, wrote the manuscript, with the interpretation of results and discussion, and carried out statistical analyses. NP contributed advising in standardization of methodology. All authors contributed to manuscript revision, read, and approved the submitted version.

## Funding

This work was supported by the Administrative Department of Science, Technology and Innovation—Colciencias (MINCIENCIAS) Contract FP44842-124-2017 and Comité para el Desarrollo de la Investigación –CODI- Universidad de Antioquia, Colombia project 2017-16341.

## Conflict of Interest

The authors declare that the research was conducted in the absence of any commercial or financial relationships that could be construed as a potential conflict of interest.

## Publisher’s Note

All claims expressed in this article are solely those of the authors and do not necessarily represent those of their affiliated organizations, or those of the publisher, the editors and the reviewers. Any product that may be evaluated in this article, or claim that may be made by its manufacturer, is not guaranteed or endorsed by the publisher.

## Abbreviations

ARG, Antibiotic resistance genes; WWTPs, wastewater treatment plants; COD, chemical oxygen demand; TOC, total organic carbon.

## References

[B1] Allen H. K. (2014). Antibiotic Resistance Gene Discovery in Food-Producing Animals. Curr. Opin. Microbiol. 19, 25–29. 10.1016/j.mib.2014.06.001 24994584

[B2] Aminov R. I. Garrigues-Jeanjean N. Mackie R. I. (2001). Molecular Ecology of Tetracycline Resistance: Development and Validation of Primers for Detection of Tetracycline Resistance Genes Encoding Ribosomal Protection Proteins. Appl. Environ. Microbiol. 67, 22–32. 10.1128/AEM.67.1.22-32.2001 11133424 PMC92507

[B3] APHA AWWA WEF. (2017). Standard Methods for the Examination of Water and Wastewater. 23rd Edition (Washington, D.C: American Public Health Association, American Water Works Association, Water Environment Federation).

[B4] Aristizábal-Hoyos A. M. Rodríguez E. A. Arias L. Jiménez J. N. (2019). High Clonal Diversity of Multidrug-Resistant and Extended Spectrum Beta-Lactamase-Producing Escherichia Coli in a Wastewater Treatment Plant. J. Environ. Manage. 245, 37–47. 10.1016/j.jenvman.2019.05.073 31150908

[B5] Arsand J. B. Hoff R. B. Jank L. Bussamara R. Dallegrave A. Bento F. M.. (2020). Presence of Antibiotic Resistance Genes and its Association With Antibiotic Occurrence in Dilúvio River in Southern Brazil. Sci. Total Environ. 738, 139781. 10.1016/j.scitotenv.2020.139781 32526421

[B6] Berendonk T. U. Manaia C. M. Merlin C. Fatta-Kassinos D. Cytryn E. Walsh F.. (2015). Tackling Antibiotic Resistance: The Environmental Framework. Nat. Rev. Microbiol. 13, 310–317. 10.1038/nrmicro3439 25817583

[B7] Berglund B. Fick J. Lindgren P.-E. (2015). Urban Wastewater Effluent Increases Antibiotic Resistance Gene Concentrations in a Receiving Northern European River. Environ. Toxicol. Chem. 34, 192–196. 10.1002/etc.2784 25331227

[B8] Botero-Coy A. M. Martínez-Pachón D. Boix C. Rincón R. J. Castillo N. Arias-Marín L. P.. (2018). ‘An Investigation Into the Occurrence and Removal of Pharmaceuticals in Colombian Wastewater’. Sci. Total Environ. 642, 842–853. 10.1016/j.scitotenv.2018.06.088 30045524

[B9] Bueno I. Travis D. Gonzalez-Rocha G. Alvarez J. Lima C. Benitez C. G.. (2019). Antibiotic Resistance Genes in Freshwater Trout Farms in a Watershed in Chile. J. Environ. Qual. 48, 1462–1471. 10.2134/jeq2018.12.0431 31589726

[B10] Bueno I. Verdugo C. Jimenez-Lopez O. Alvarez P. P. Gonzalez-Rocha G. Lima C. A.. (2020). Role of Wastewater Treatment Plants on Environmental Abundance of Antimicrobial Resistance Genes in Chilean Rivers. Int. J. Hyg. Environ. Health 223, 56–64. 10.1016/j.ijheh.2019.10.006 31722832

[B11] Cacace D. Fatta-Kassinos D. Manaia C. M. Cytryn E. Kreuzinger N. Rizzo L.. (2019). Antibiotic Resistance Genes in Treated Wastewater and in the Receiving Water Bodies: A Pan-European Survey of Urban Settings. Water Res. 162, 320–330. 10.1016/j.watres.2019.06.039 31288142

[B12] Calero-Cáceres W. Melgarejo A. Colomer-Lluch M. Stoll C. Lucena F. Jofre J.. (2014). Sludge as a Potential Important Source of Antibiotic Resistance Genes in Both the Bacterial and Bacteriophage Fractions. Environ. Sci. Technol. 48, 7602–7611. 10.1021/es501851s 24873655

[B13] Caucci S. Karkman A. Cacace D. Rybicki M. Timpel P. Voolaid V.. (2016). Seasonality of Antibiotic Prescriptions for Outpatients and Resistance Genes in Sewers and Wastewater Treatment Plant Outflow. FEMS Microbiol. Ecol. 92, fiw060. 10.1093/femsec/fiw060 27073234

[B14] Chagas T. P. G. Seki L. M. da Silva D. M. Asensi M. D. (2011). Occurrence of KPC-2-Producing Klebsiella Pneumoniae Strains in Hospital Wastewater. J. Hosp. Infect. 77, 281. 10.1016/j.jhin.2010.10.008 21216031

[B15] Czekalski N. Berthold T. Caucci S. Egli A. Bürgmann H. (2012). Increased Levels of Multiresistant Bacteria and Resistance Genes After Wastewater Treatment and Their Dissemination Into Lake Geneva, Switzerland. Front. Microbiol. 3, 106. 10.3389/fmicb.2012.00106 22461783 PMC3310248

[B16] de Oliveira D. V. Nunes L. S. Barth A. L. van der Sand S. T. (2017). Genetic Background of β-Lactamases in Enterobacteriaceae Isolates From Environmental Samples. Microb. Ecol. 74, 599–607. 10.1007/s00248-017-0970-6 28378066

[B17] Di Cesare A. Eckert E. M. Rogora M. Corno G. (2017). Rainfall Increases the Abundance of Antibiotic Resistance Genes Within a Riverine Microbial Community. Environ. Pollut. 226, 473–478. 10.1016/j.envpol.2017.04.036 28438356

[B18] Gillings M. R. (2014). Integrons: Past, Present, and Future. Microbiol. Mol. Biol. Rev. 78, 257–277. 10.1128/MMBR.00056-13 24847022 PMC4054258

[B19] Girardi R. Pinheiro A. Garbossa L. H. P. Torres É. (2016). Water Quality Change of Rivers During Rainy Events in a Watershed With Different Land Uses in Southern Brazil. RBRH 21, 514–524. 10.1590/2318-0331.011615179

[B20] Hall C. B. Lipton R. B. Sliwinski M. Stewart W. F. (2000). A Change Point Model for Estimating the Onset of Cognitive Decline in Preclinical Alzheimer’s Disease. Stat. Med. 19, 1555–1566. 10.1002/(sici)1097-0258(20000615/30)19:11/12<1555::aid-sim445>3.0.co;2-3 10844718

[B21] Hernando-Amado S. Coque T. M. Baquero F. Martínez J. L. (2019). Defining and Combating Antibiotic Resistance From One Health and Global Health Perspectives. Nat. Microbiol. 4, 1432–1442. 10.1038/s41564-019-0503-9 31439928

[B22] Hong P.-Y. Al-Jassim N. Ansari M. Mackie R. (2013). Environmental and Public Health Implications of Water Reuse: Antibiotics, Antibiotic Resistant Bacteria, and Antibiotic Resistance Genes. Antibiotics 2, 367–399. 10.3390/antibiotics2030367 27029309 PMC4790270

[B23] IDEAM - Instituto de Hidrología, M. y E. A. (2017). IDEAM - Instituto De Hidrología, Meteorología Y Estudios Ambientales. Predicc. Clim. Available at: http://www.ideam.gov.co/web/tiempo-y-clima/clima (Accessed August 10, 2017).

[B24] Kim S. Jensen J. N. Aga D. S. Weber A. S. (2007). Tetracycline as a Selector for Resistant Bacteria in Activated Sludge. Chemosphere 66, 1643–1651. 10.1016/j.chemosphere.2006.07.066 16959296

[B25] Koczura R. Mokracka J. Taraszewska A. Łopacinska N. (2016). Abundance of Class 1 Integron-Integrase and Sulfonamide Resistance Genes in River Water and Sediment Is Affected by Anthropogenic Pressure and Environmental Factors. Microb. Ecol. 72, 909–916. 10.1007/s00248-016-0843-4 27599709 PMC5080314

[B26] Korzeniewska E. Harnisz M. (2018). Relationship Between Modification of Activated Sludge Wastewater Treatment and Changes in Antibiotic Resistance of Bacteria. Sci. Total Environ. 639, 304–315. 10.1016/j.scitotenv.2018.05.165 29791883

[B27] Lamba M. Ahammad S. Z. (2017). Sewage Treatment Effluents in Delhi: A Key Contributor of β-Lactam Resistant Bacteria and Genes to the Environment. Chemosphere 188, 249–256. 10.1016/j.chemosphere.2017.08.133 28886559

[B28] Lee C. Kim J. Shin S. G. Hwang S. (2006). Absolute and Relative QPCR Quantification of Plasmid Copy Number in Escherichia Coli. J. Biotechnol. 123, 273–280. 10.1016/j.jbiotec.2005.11.014 16388869

[B29] Leon-Felix J. Esperón F. Adell A. D. Moreno-Switt A. I. Rivera D. Caipo M. L.. (2020). Antimicrobial Resistance in Water in Latin America and the Caribbean: Available Research and Gaps. Front. Vet. Sci. 7, 546 10.3389/fvets.2020.00546

[B30] Makowska N. Koczura R. Mokracka J. (2016). Class 1 Integrase, Sulfonamide and Tetracycline Resistance Genes in Wastewater Treatment Plant and Surface Water. Chemosphere 144, 1665–1673. 10.1016/j.chemosphere.2015.10.044 26519797

[B31] Manaia C. M. Rocha J. Scaccia N. Marano R. Radu E. Biancullo F.. (2018). Antibiotic Resistance in Wastewater Treatment Plants: Tackling the Black Box. Environ. Int. 115, 312–324. 10.1016/j.envint.2018.03.044 29626693

[B32] Mao D. Yu S. Rysz M. Luo Y. Yang F. Li F.. (2015). Prevalence and Proliferation of Antibiotic Resistance Genes in Two Municipal Wastewater Treatment Plants. Water Res. 85, 458–466. 10.1016/j.watres.2015.09.010 26372743

[B33] Marti E. Balcázar J. L. (2013). Real-Time PCR Assays for Quantification of Qnr Genes in Environmental Water Samples and Chicken Feces. Appl. Environ. Microbiol. 79, 1743–1745. 10.1128/AEM.03409-12 23275512 PMC3591933

[B34] Marti E. Jofre J. Balcazar J. L. (2013). Prevalence of Antibiotic Resistance Genes and Bacterial Community Composition in a River Influenced by a Wastewater Treatment Plant. PloS One 8, e78906. 10.1371/journal.pone.0078906 24205347 PMC3808343

[B35] Milaković M. Vestergaard G. González-Plaza J. J. Petrić I. Šimatović A. Senta I.. (2019). Pollution From Azithromycin-Manufacturing Promotes Macrolide-Resistance Gene Propagation and Induces Spatial and Seasonal Bacterial Community Shifts in Receiving River Sediments. Environ. Int. 123, 501–511. 10.1016/j.envint.2018.12.050 30622075

[B36] Ministry of Environment and Sustainable Development Colombia. Resolución 363/2015. de los parámetros y los valores límites máximos permisibles en los vertimientos puntuales a cuerpos de aguas superficiales y a los sistemas de alcantarillado público y se dictan otras disposiciones. Available at: https://www.minambiente.gov.co/images/normativa/app/resoluciones/d1-res_631_marz_2015.pdf.

[B37] Mojica M. F. Correa A. Vargas D. A. Maya J. J. Montealegre M. C. Rojas L. J.. (2012). Molecular Correlates of the Spread of KPC-Producing Enterobacteriaceae in Colombia. Int. J. Antimicrob. Agents 40, 277–279. 10.1016/j.ijantimicag.2012.05.006 22789725

[B38] Munoz-Price L. S. Poirel L. Bonomo R. A. Schwaber M. J. Daikos G. L. Cormican M.. (2013). Clinical Epidemiology of the Global Expansion of Klebsiella Pneumoniae Carbapenemases. Lancet Infect. Dis. 13, 785–796. 10.1016/S1473-3099(13)70190-7 23969216 PMC4673667

[B39] Novo A. André S. Viana P. Nunes O. C. Manaia C. M. (2013). Antibiotic Resistance, Antimicrobial Residues and Bacterial Community Composition in Urban Wastewater. Water Res. 47, 1875–1887. 10.1016/j.watres.2013.01.010 23375783

[B40] Ocampo A. M. Chen L. Cienfuegos A. V. Roncancio G. Chavda K. D. Kreiswirth B. N.. (2016). A Two-Year Surveillance in Five Colombian Tertiary Care Hospitals Reveals High Frequency of Non-CG258 Clones of Carbapenem-Resistant Klebsiella Pneumoniae With Distinct Clinical Characteristics. Antimicrob. Agents Chemother. 60, 332–342. 10.1128/AAC.01775-15 26503660 PMC4704171

[B41] Pallares-Vega R. Blaak H. van der Plaats R. de Roda Husman A. M. Hernandez Leal L. van Loosdrecht M. C. M. (2019). Determinants of Presence and Removal of Antibiotic Resistance Genes During WWTP Treatment: A Cross-Sectional Study. Water Res. 161, 319–328. 10.1016/j.watres.2019.05.100 31203037

[B42] Paulus G. K. Hornstra L. M. Medema G. (2020). International Tempo-Spatial Study of Antibiotic Resistance Genes Across the Rhine River Using Newly Developed Multiplex qPCR Assays. Sci. Total Environ. 706, 135733. 10.1016/j.scitotenv.2019.135733 31818563

[B43] Pei R. Kim S.-C. Carlson K. H. Pruden A. (2006). Effect of River Landscape on the Sediment Concentrations of Antibiotics and Corresponding Antibiotic Resistance Genes (ARG). Water Res. 40, 2427–2435. 10.1016/j.watres.2006.04.017 16753197

[B44] Picão R. C. Cardoso J. P. Campana E. H. Nicoletti A. G. Petrolini F. V. Assis D. M.. (2013). The Route of Antimicrobial Resistance From the Hospital Effluent to the Environment: Focus on the Occurrence of KPC-Producing Aeromonas Spp. And Enterobacteriaceae in Sewage. Diagn. Microbiol. Infect. Dis. 76, 80–85. 10.1016/j.diagmicrobio.2013.02.001 23478032

[B45] Poirel L. Cattoir V. Nordmann P. (2008). Is Plasmid-Mediated Quinolone Resistance a Clinically Significant Problem? Clin. Microbiol. Infect. 14, 295–297. 10.1111/j.1469-0691.2007.01930.x 18190576

[B46] Rafraf I. D. Lekunberri I. Sànchez-Melsió A. Aouni M. Borrego C. M. Balcázar J. L. (2016). Abundance of Antibiotic Resistance Genes in Five Municipal Wastewater Treatment Plants in the Monastir Governorate, Tunisia. Environ. Pollut. 219, 353–358. 10.1016/j.envpol.2016.10.062 27814552

[B47] Rodríguez E. A. Aristizábal-Hoyos A. M. Morales-Zapata S. Arias L. Jiménez J. N. (2020). High Frequency of Gram-Negative Bacilli Harboring blaKPC-2 in the Different Stages of Wastewater Treatment Plant: A Successful Mechanism of Resistance to Carbapenems Outside the Hospital Settings. J. Environ. Manage. 271, 111046. 10.1016/j.jenvman.2020.111046 32778323

[B48] Rodriguez-Mozaz S. Chamorro S. Marti E. Huerta B. Gros M. Sànchez-Melsió A.. (2015). Occurrence of Antibiotics and Antibiotic Resistance Genes in Hospital and Urban Wastewaters and Their Impact on the Receiving River. Water Res. 69, 234–242. 10.1016/j.watres.2014.11.021 25482914

[B49] Santamaría J. (2011). Detection and Diversity Evaluation of Tetracycline Resistance Genes in Grassland-Based Production Systems in Colombia, South America. Front. Microbiol. 2, 252. 10.3389/fmicb.2011.00252 22174707 PMC3237277

[B50] Schages L. Wichern F. Kalscheuer R. Bockmühl D. (2020). Winter is Coming - Impact of Temperature on the Variation of Beta-Lactamase and Mcr Genes in a Wastewater Treatment Plant. Sci. Total Environ. 712, 136499. 10.1016/j.scitotenv.2020.136499 31945531

[B51] Sims N. Kasprzyk-Hordern B. (2020). Future Perspectives of Wastewater-Based Epidemiology: Monitoring Infectious Disease Spread and Resistance to the Community Level. Environ. Int. 139, 105689. 10.1016/j.envint.2020.105689 32283358 PMC7128895

[B52] Sui Q. Zhang J. Tong J. Chen M. Wei Y. (2017). Seasonal Variation and Removal Efficiency of Antibiotic Resistance Genes During Wastewater Treatment of Swine Farms. Environ. Sci. Pollut. Res. 24, 9048–9057. 10.1007/s11356-015-5891-7 26715413

[B53] van den Hout A. Muniz-Terrera G. Matthews F. E. (2013). Change Point Models for Cognitive Tests Using Semi-Parametric Maximum Likelihood. Comput. Stat. Data Anal. 57, 684–698. 10.1016/j.csda.2012.07.024 23471297 PMC3587404

[B54] Vaz-Moreira I. Varela A. R. Pereira T. V. Fochat R. C. Manaia C. M. (2016). Multidrug Resistance in Quinolone-Resistant Gram-Negative Bacteria Isolated From Hospital Effluent and the Municipal Wastewater Treatment Plant. Microb. Drug Resist. 22, 155–163. 10.1089/mdr.2015.0118 26469134

[B55] Virginia Villegas M. Lolans K. Correa A. Jose Suarez C. Lopez J. A. Vallejo M.. (2006). First Detection of the Plasmid-Mediated Class A Carbapenemase KPC-2 in Clinical Isolates of Klebsiella Pneumoniae From South America. Antimicrob. Agents Chemother. 50, 2880–2882. 10.1128/AAC.00186-06 16870793 PMC1538657

[B56] Wang Z. Zhang X. X. Huang K. Miao Y. Shi P. Liu B.. (2013). Metagenomic Profiling of Antibiotic Resistance Genes and Mobile Genetic Elements in a Tannery Wastewater Treatment Plant. PloS One 8, 9. 10.1371/journal.pone.0076079 PMC3787945 24098424

[B57] Xiong W. Sun Y. Ding X. Zhang Y. Zeng Z. (2014). Antibiotic Resistance Genes Occurrence and Bacterial Community Composition in the Liuxi River. Front. Environ. Sci. 2, 61. 10.3389/fenvs.2014.00061

[B58] Yang F. Huang L. Li L. Yang Y. Mao D. Luo Y. (2017). Discharge of KPC-2 Genes From the WWTPs Contributed to Their Enriched Abundance in the Receiving River. Sci. Total Environ. 581–582, 136–143. 10.1016/j.scitotenv.2016.12.063 28065546

[B59] Yang F. Mao D. Zhou H. Luo Y. (2016). Prevalence and Fate of Carbapenemase Genes in a Wastewater Treatment Plant in Northern China. PloS One 11, e0156383. 10.1371/journal.pone.0156383 27227329 PMC4882038

[B60] Yang Y. Li B. Ju F. Zhang T. (2013). Exploring Variation of Antibiotic Resistance Genes in Activated Sludge Over a Four-Year Period Through a Metagenomic Approach. Env. Sci. Technol. 47, 10197–10205. 10.1021/es4017365 23919449

