# Supplementary material for: Climatological and Epidemiological Conditions Are Important Factors Related to the Abundance of bla KPC and Other Antibiotic Resistance Genes (ARGs) in Wastewater Treatment Plants and Their Effluents, in an Endemic Country
Source: Front Cell Infect Microbiol. 2021 Aug 13;11:686472. doi: 10.3389/fcimb.2021.686472 (PMC8414572; doi:10.3389/fcimb.2021.686472)
Supplement: Supplementary file 1 [file DataSheet_1.docx]

Supplementary Material

# Supplementary Figures and Tables

**Table S.1**. Primers used in this study

| **Target gene** | **Primers** | **Sequence** | **Conditions** | **Reference** |
| --- | --- | --- | --- | --- |
| *16S rRNA* | F1048 | GTGSTGCAYGGYTGTCGTCA | 95ºC 3 min (1 cycle); 95ºC 15 s, 60ºC 1 min s and 72ºC 30 s (35 cycles) | (Maeda et al., 2003) |
|  | R1194 | ACGTCRTCCMCACCTTCCTC |  |  |
| *bla*_CTX-M_ | RTCTXM-F | CTATGGCACCACCAACGATA | 95ºC 3 min (1 cycle); 95ºC 15 s, 60ºC 20 s and 72ºC 30 s (40 cycles) | (Marti et al., 2013) |
|  | RTCTXM-R | ACGGCTTTCTGCCTTAGGTT |  |  |
| *bla*_SHV_ | RTblaSHVF | CGCTTTCCCATGATGAGCACCTTT | 95ºC 3 min (1 cycle); 95ºC 15 s, 64ºC 30 s and 72ºC 30 s (40 cycles) | (Xi et al., 2009) |
|  | RTblaSHVR | TCCTGCTGGCGATAGTGGATCTTT |  |  |
| *erm*B | erm(B)-91f | GATACCGTTTACGAAATTGG | 95ºC 3 min (1 cycle); 95ºC 15 s, 58ºC 20 s and 72ºC 30 s (40 cycles) | (Chen et al., 2007) |
|  | erm(B)-454r | GAATCGAGACTTGAGTGTGC |  |  |
| *qnr*A | qnrA-R | ATTTCTCACGCCAGGATTTG | 95ºC 3 min (1 cycle); 95ºC 15 s, 64ºC 20 s and 72ºC 30 s (40 cycles) | (Robicsek et al., 2006; Marti and Balcázar, 2013) |
|  | qnrA-F | GATCGGCAAAGGTTAGGTCA |  |  |
| *sul*1 | Sul(I)-FW | CGCACCGGAAACATCGCTGCAC | 95ºC 3 min (1 cycle); 95ºC 15 s, 65ºC 20 s and 72ºC 30 s (40 cycles) | (Pei et al., 2006) |
|  | Sul(I)-RV | TGAAGTTCCGCCGCAAGGCTCG |  |  |
| *sul*2 | Sul(II)-FW | TCCGGTGGAGGCCGGTATCTGG | 95ºC 3 min (1 cycle); 95ºC 15 s, 58ºC 20 s and 72ºC 30 s (40 cycles) | (Pei et al., 2006) |
|  | Sul(II)-RV | CGGGAATGCCATCTGCCTTGAG |  |  |
| *tet*W | tet(W)-FW | GAGAGCCTGCTATATGCCAGC | 95ºC 3 min (1 cycle); 95ºC 15 s, 60ºC 20 s and 72ºC 30 s (40 cycles) | (Aminov et al., 2001) |
|  | tet(W)-RV | GGGCGTATCCACAATGTTAAC |  |  |
| *bla*_NDM_ | Ndm-rtF | GATTGCGACTTATGCCAATG | 95ºC 3 min (1 cycle); 95ºC 30 s, 60°C 1 min and 72ºC 30 s (40 cycles) | (Subirats et al., 2017) |
|  | Ndm-rtR | TCGATCCCAACGGTGATATT |  |  |
| *bla*_OXA48_ | Oxa-rtF | AGGCACGTATGAGCAAGATG | 95ºC 5 min (1 cycle); 95ºC 15 s, 47°C 1 min and 72ºC 30 s (40 cycles) | (Subirats et al., 2017) |
|  | Oxa-rtR | TGGCTTGTTTGACAATACGC |  |  |
| *bla*_KPC2_ | SK-FW | GCTTCCCACTGTGCAGCTCATTC | 95ºC 5 min (1 cycle); 95ºC 15 s, 66,1°C 1 min and 72ºC 30 s (40 cycles) | (Yang et al., 2016) |
|  | SK-RV | CGCCCAACTCCTTCAGCAACAAATTG |  |  |

**Table S.2.** Absolute abundance of antibiotic resistance genes (copies per ml) through WWTP

(*) denotes a statistically significant difference between points (P<0.05). SD: Standard Deviation, *b.d.l:* below detection limit**Table S.3 Number of ARGS among samples taken from the WWTP (n=44).**

| **Gene** | **Raw influent (RI) (n=11)** | **Aeration Tank  (AeT) (n=11)** | **Return actived sludge (RS) (n=11)** | **Final effluent (FE) (n=11)** | **Total (n=44)** |
| --- | --- | --- | --- | --- | --- |
|  |  |  |  |  |  |
|  |  |  |  |  |  |
| ***16S rRNA*** | 11 | 11 | 11 | 11 | 44 |
| ***bla*_KPC_** | 11 | 11 | 11 | 11 | 44 |
| ***bla*_NDM_** | 8 | 1 | 3 | 3 | 15 |
| ***bla*_OXA-48_** | 11 | 7 | 7 | 11 | 36 |
| ***bla*_CTX-M_** | 11 | 11 | 11 | 11 | 44 |
| ***bla*_SHV_** | 11 | 9 | 5 | 11 | 36 |
| ***erm*B** | 9 | 8 | 11 | 8 | 36 |
| ***su*l1** | 11 | 11 | 11 | 11 | 44 |
| ***sul*2** | 11 | 11 | 11 | 11 | 44 |
| ***tet*W** | 11 | 11 | 11 | 11 | 44 |
| ***qnr*A** | 9 | 4 | 8 | 10 | 31 |

**Table S4. ARGs removal observed in this study**

| **Gene** | **Aeration Tank** | | **Return actived sludge** | | **Final effluent** | |
| --- | --- | --- | --- | --- | --- | --- |
|  | **Mean (SD)** | | **Mean (SD)** | | **Mean (SD)** | |
| ***16S rRNA*** | 0,44 | (0,62) | 0,27 | (0,25) | 0,10 | (0,12) |
| ***bla*_KPC_** | 1,42 | (1,64) | 0,54 | (0,23) | 0,02 | (0,19) |
| ***bla*_NDM_** | 1,10 | (0,00) | 0,67 | (0,37) | 0,29 | (-0,03) |
| ***bla*_OXA-48_** | 1,15 | (1,53) | 1,22 | (1,41) | **-0,46** | (-0,46) |
| ***bla*_CTX-M_** | 1,85 | (2,04) | 0,44 | (0,05) | 0,26 | (0,35) |
| ***bla*_SHV_** | 1,52 | (1,99) | 1,37 | (1,27) | 0,36 | (0,61) |
| ***erm*B** | 1,30 | (1,31) | 0,32 | (**-0,24**) | 0,29 | (0,20) |
| ***sul*1** | 0,52 | (0,83) | 0,28 | (0,47) | 0,06 | (0,28) |
| ***sul*2** | 0,42 | (0,44) | 0,00 | (0,08) | **-0,08** | (0,00) |
| ***tet*W** | 0,84 | (1,25) | 0,62 | (0,58) | 0,28 | (0,49) |
| ***qnr*A** | 0,94 | (1,63) | 0,95 | (1,28) | 0,40 | (0,70) |

Values are expressed in log_10_. An estimation of the removal values was calculated (Average log reduction = log_10_ (mean gen copies influent) /( mean gen copies in respective WWTP stage)). Abbreviations: SD: Standard Deviation

**Table S.5. Statistic estimation by Spearman or Pearson correlation between ARG counts and physico-chemical parameters and atmospheric conditions**

* Person. A significant difference (p≤0.05) are shown in dark gray and bold.

**Table S.6. Mixed-effects generalized linear model. Bivariate analyses: factors related to the abundance of *bla*_KPC_ in the WWTP during the study time.**

| **Factor** | **Crude** | | | |
| --- | --- | --- | --- | --- |
|  | **β** | **p value** | **CI 95%** | |
| **Season** |  |  |  | |
| **Rainy** | -0,705 | **0,146** | -1,656 | 0,245 |
| **Water temperature (°C)** | -0,057 | 0,772 | -0,443 | 0,329 |
| **pH** | -0,130 | 0,625 | -0,650 | 0,391 |
| **Conductivity (μS/cm)** | 0,001 | 0,278 | -0,001 | 0,003 |
| **Dissolved Oxygen (mg/L)** | 0,746 | **0,000** | 0,390 | 1,103 |
| **TOC (mg/L)** | -0,001 | 0,759 | -0,008 | 0,006 |
| **COD (mgO_2_/L)** | 0,001 | 0,614 | -0,001 | 0,002 |
| **Total solids (mg/L)** | 0,000 | 0,853 | -0,002 | 0,002 |
| **Precipitation one day before of sampling (mm)** | -0,011 | 0,541 | -0,047 | 0,024 |
| **Precipitation in the sampling day (mm)** | 0,018 | 0,630 | -0,055 | 0,090 |

A significant difference (p≤0.25) are shown in dark gray and bold.

**Table S.7. Model 1. Mixed-effects generalized linear model. Factors related to the abundance of *bla*_KPC_ in the WWTP during the study time.**

| **Factor** | **Crude** | | | | **Adjusted** | | | |
| --- | --- | --- | --- | --- | --- | --- | --- | --- |
|  | **β** | **p value** | **CI 95%** | | **β** | **p value** | **CI 95%** | |
| Season |  |  |  | |  |  |  |  |
| Rainy | -0,705 | **0,146** | -1,656 | 0,245 | 0,497 | 0,194 | -0,253 | 1,248 |
| Dissolved Oxygen (mg/L) | 0,746 | **0,000** | 0,390 | 1,103 | 0,716 | **0,000** | 0,337 | 1,094 |

Multilevel mixed-effects generalized linear model (GLM). Gama, β:model estimate. Adjusted by sampling time.

## Supplementary Figures

**
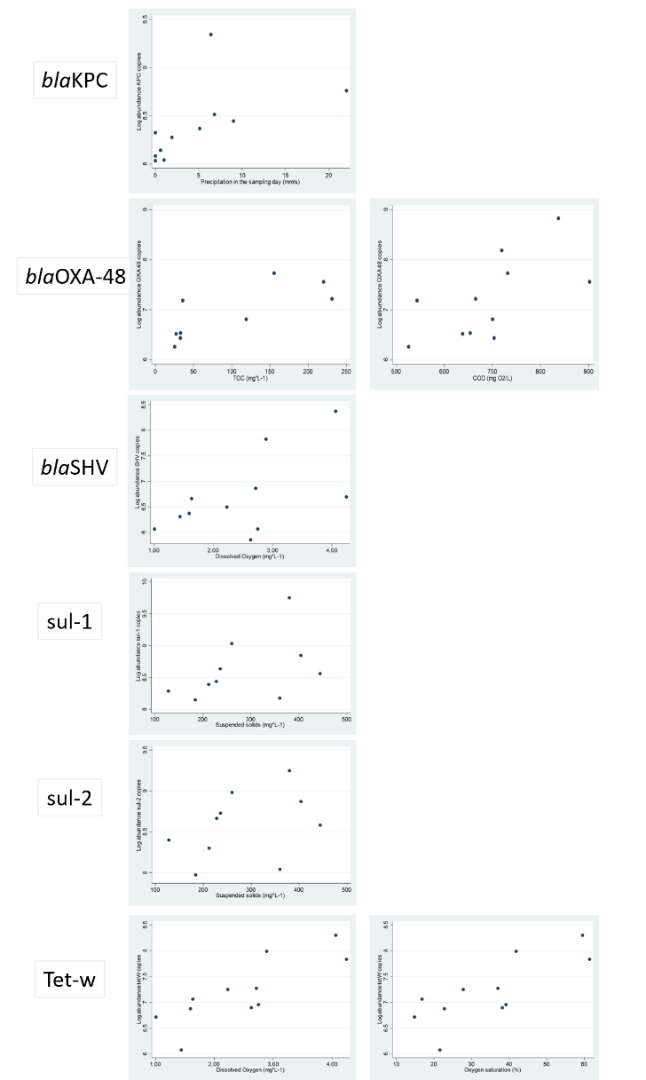
**

**Supplementary Figure 1.** Statistic estimation by Spearman or Pearson correlation between ARG counts and physicochemical parameters in the Raw Influent. The figure shows those variables in which correlations were detected.


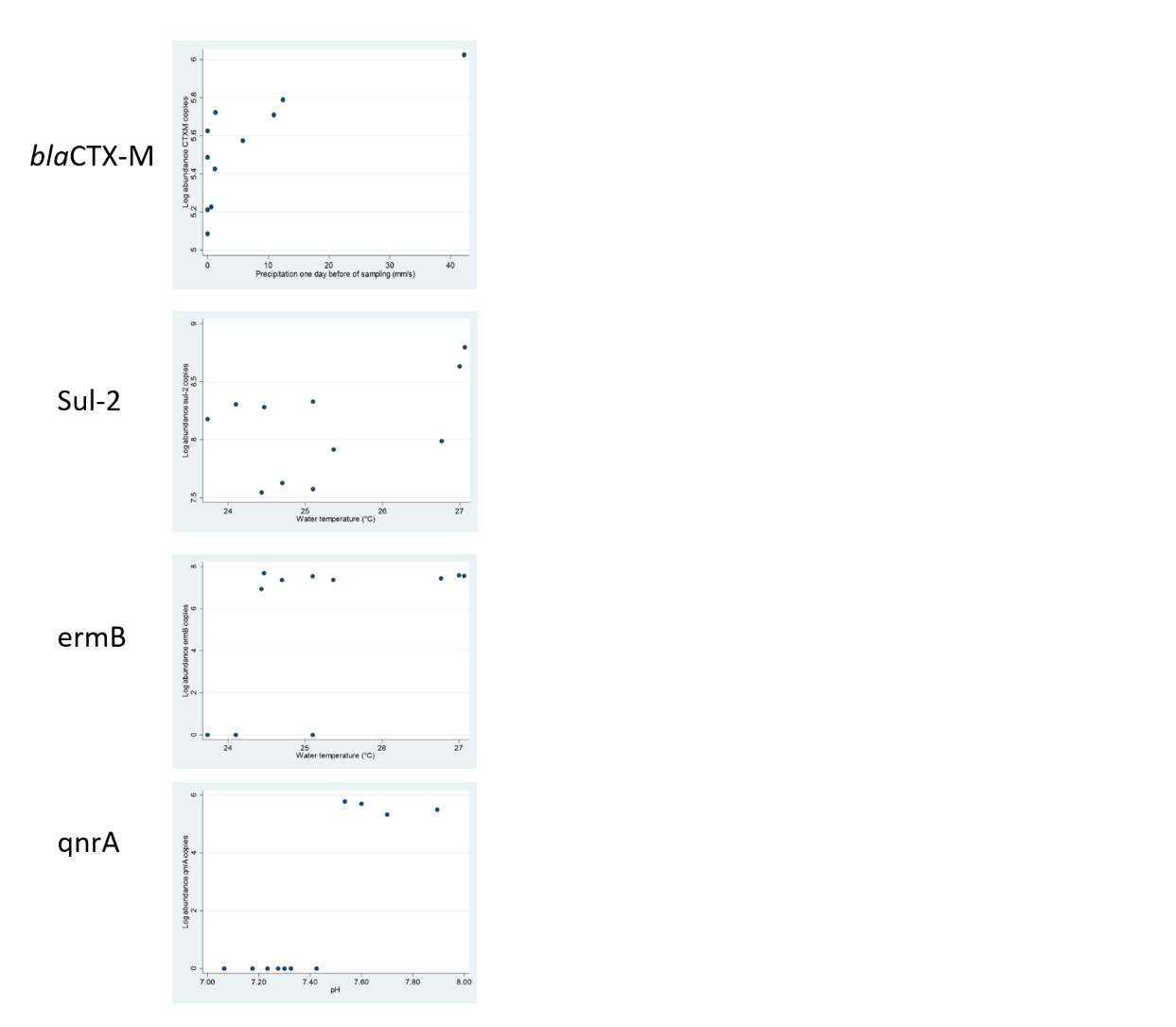


**Supplementary Figure 2.** Statistic estimation by Spearman or Pearson correlation between ARG counts and physicochemical parameters in Aeration Tank**.** The figure shows those variables in which correlations were detected.


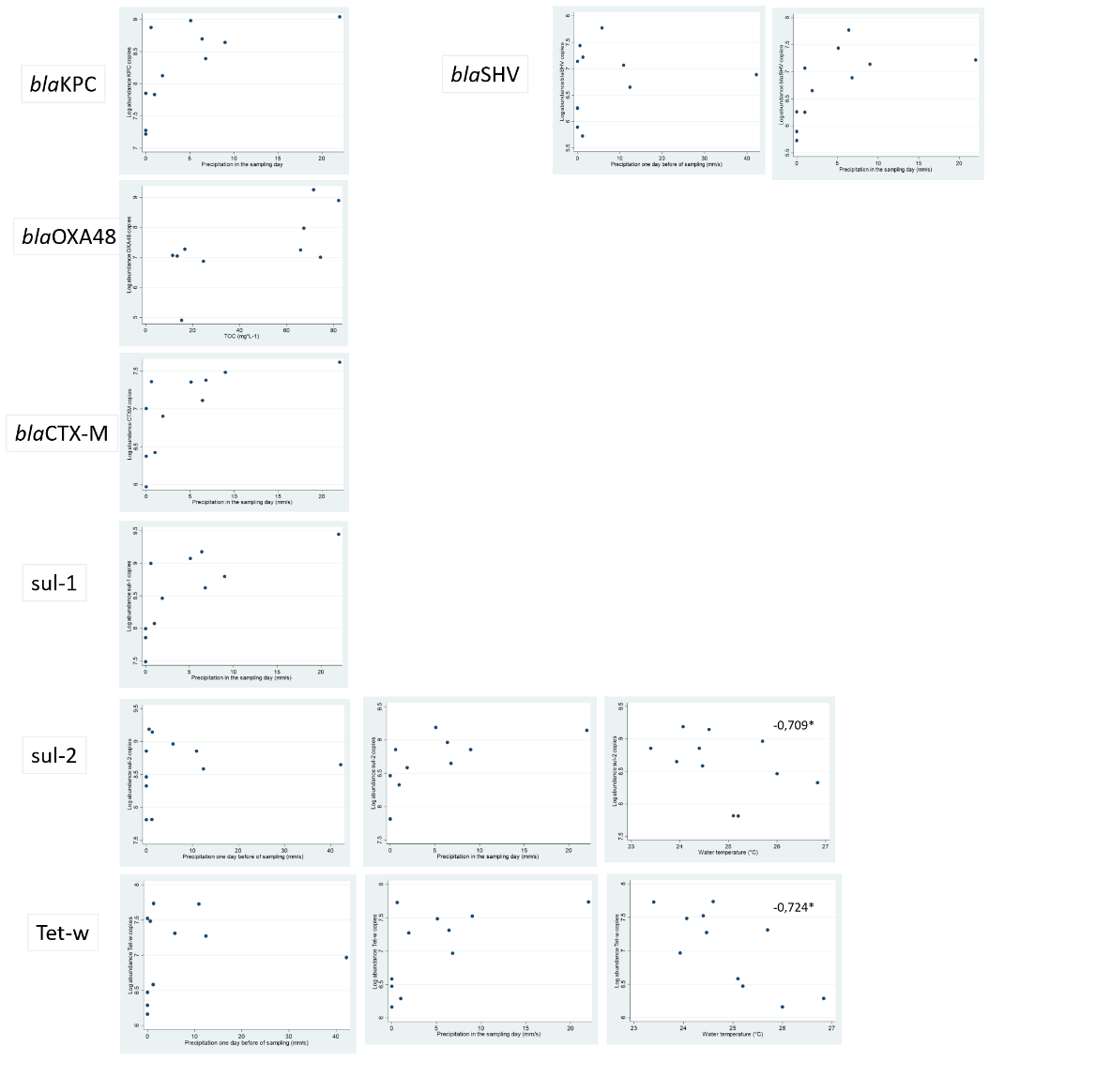


**Supplementary Figure 3.** Statistic estimation by Spearman or Pearson correlation between ARGs counts and physicochemical parameters in final effluent**.** The figure shows those variables in which correlations were detected.


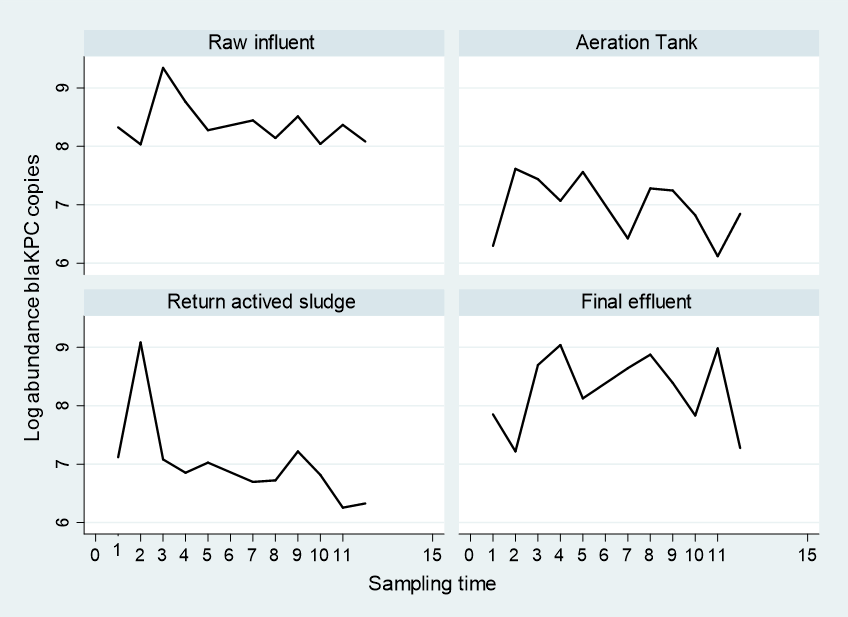


**Supplementary Figure 4.** Distribution of *bla*_KPC_ over the study period by sampling place

**References**

Aminov, R. I., Garrigues-Jeanjean, N., and Mackie, R. I. (2001). Molecular ecology of tetracycline resistance: Development and validation of primers for detection of tetracycline resistance genes encoding ribosomal protection proteins. *Appl. Environ. Microbiol.* 67, 22–32. doi:10.1128/AEM.67.1.22-32.2001.

Chen, J., Yu, Z., Michel, F. C., Wittum, T., and Morrison, M. (2007). Development and application of real-time PCR assays for quantification of erm genes conferring resistance to macrolides-lincosamides-streptogramin B in livestock manure and manure management systems. *Appl. Environ. Microbiol.* 73, 4407–16. doi:10.1128/AEM.02799-06.

Lee, C., Kim, J., Shin, S. G., and Hwang, S. (2006). Absolute and relative QPCR quantification of plasmid copy number in Escherichia coli. *J. Biotechnol.* 123, 273–80. doi:10.1016/j.jbiotec.2005.11.014.

Maeda, H., Fujimoto, C., Haruki, Y., Maeda, T., Kokeguchi, S., Petelin, M., et al. (2003). Quantitative real-time PCR using TaqMan and SYBR Green for Actinobacillus actinomycetemcomitans , Porphyromonas gingivalis , Prevotella intermedia , tetQ gene and total bacteria. *FEMS Immunol. Med. Microbiol.* 39, 81–86. doi:10.1016/S0928-8244(03)00224-4.

Marti, E., and Balcázar, J. L. (2013). Antibiotic resistance in the aquatic environment. *Compr. Anal. Chem.* 62, 671–684. doi:10.1016/B978-0-444-62657-8.00019-7.

Marti, E., Jofre, J., and Balcazar, J. L. (2013). Prevalence of Antibiotic Resistance Genes and Bacterial Community Composition in a River Influenced by a Wastewater Treatment Plant. *PLoS One* 8, e78906. doi:10.1371/journal.pone.0078906.

Pei, R., Kim, S.-C., Carlson, K. H., and Pruden, A. (2006). Effect of River Landscape on the sediment concentrations of antibiotics and corresponding antibiotic resistance genes (ARG). *Water Res.* 40, 2427–2435. doi:10.1016/j.watres.2006.04.017.

Robicsek, A., Strahilevitz, J., Sahm, D. F., Jacoby, G. A., and Hooper, D. C. (2006). qnr prevalence in ceftazidime-resistant Enterobacteriaceae isolates from the United States. *Antimicrob. Agents Chemother.* 50, 2872–4. doi:10.1128/AAC.01647-05.

Subirats, J., Royo, E., Balcázar, J. L., and Borrego, C. M. (2017). Real-time PCR assays for the detection and quantification of carbapenemase genes (bla KPC, bla NDM, and bla OXA-48) in environmental samples. *Environ. Sci. Pollut. Res.* 24, 6710–6714. doi:10.1007/s11356-017-8426-6.

Xi, C., Zhang, Y., Marrs, C. F., Ye, W., Simon, C., Foxman, B., et al. (2009). Prevalence of Antibiotic Resistance in Drinking Water Treatment and Distribution Systems. *Appl. Environ. Microbiol.* 75, 5714–5718. doi:10.1128/AEM.00382-09.

Yang, F., Mao, D., Zhou, H., and Luo, Y. (2016). Prevalence and Fate of Carbapenemase Genes in a Wastewater Treatment Plant in Northern China. *PLoS One* 11, e0156383. doi:10.1371/journal.pone.0156383.
